# Schizophrenia-related microdeletion causes defective ciliary motility and brain ventricle enlargement via microRNA-dependent mechanisms in mice

**DOI:** 10.1038/s41467-020-14628-y

**Published:** 2020-02-14

**Authors:** Tae-Yeon Eom, Seung Baek Han, Jieun Kim, Jay A. Blundon, Yong-Dong Wang, Jing Yu, Kara Anderson, Damian B. Kaminski, Sadie Miki Sakurada, Shondra M. Pruett-Miller, Linda Horner, Ben Wagner, Camenzind G. Robinson, Matthew Eicholtz, Derek C. Rose, Stanislav S. Zakharenko

**Affiliations:** 1 Department of Developmental Neurobiology, St. Jude Children’s Research Hospital, Memphis, TN 38105 USA; 2 Center for In Vivo Imaging and Therapeutics, Cellular Imaging Shared Resource, St. Jude Children’s Research Hospital, Memphis, TN 38105 USA; 3 Department of Computational Biology, St. Jude Children’s Research Hospital, Memphis, TN 38105 USA; 4 Center for Advanced Genome Engineering, St. Jude Children’s Research Hospital, Memphis, TN 38105 USA; 5 Cellular Imaging Shared Resource, St. Jude Children’s Research Hospital, Memphis, TN 38105 USA; 6 Electrical and Electronics Systems Research Division, Oak Ridge National Laboratory, Oak Ridge, TN 37831 USA; 7 Present Address: Department of Computer Science, Florida Southern College, Lakeland, FL 33801 USA

**Keywords:** Cellular neuroscience, Schizophrenia

## Abstract

Progressive ventricular enlargement, a key feature of several neurologic and psychiatric diseases, is mediated by unknown mechanisms. Here, using murine models of 22q11-deletion syndrome (22q11DS), which is associated with schizophrenia in humans, we found progressive enlargement of lateral and third ventricles and deceleration of ciliary beating on ependymal cells lining the ventricular walls. The cilia-beating deficit observed in brain slices and in vivo is caused by elevated levels of dopamine receptors (Drd1), which are expressed in motile cilia. Haploinsufficiency of the microRNA-processing gene *Dgcr8* results in Drd1 elevation, which is brought about by a reduction in *Drd1*-targeting microRNAs miR-382-3p and miR-674-3p. Replenishing either microRNA in 22q11DS mice normalizes ciliary beating and ventricular size. Knocking down the microRNAs or deleting their seed sites on *Drd1* mimicked the cilia-beating and ventricular deficits. These results suggest that the Dgcr8–miR-382-3p/miR-674-3p–Drd1 mechanism contributes to deceleration of ciliary motility and age-dependent ventricular enlargement in 22q11DS.

## Abstract (web summary)

Schizophrenia is associated with brain ventricular enlargement. Here, the authors show in mice that 22q11 deletion, which is associated with schizophrenia, causes ventricular enlargement and motility abnormalities in cilia lining ventricle walls via a microRNA mechanism.

## Introduction

The volume of cerebral ventricles often increases in various neurologic and psychiatric disorders, such as Parkinson disease^1^, Alzheimer disease^2^, vascular dementia^3^, bipolar disorder^4^, copy number variation disorders^5^, as well as during normal aging^6–8^. In individuals with schizophrenia (SCZ), cerebral ventricular enlargement is one of the most common and replicable findings; it has been observed in more than 80% of SCZ studies^9–16^. The progressive increase in the total or regional ventricular volumes usually becomes evident at the first episode of disease^17,18^. Despite this strong linkage, the mechanisms of ventricular enlargement in psychiatric disorders are mostly unknown.

Many neurodevelopmental disorders, such as SCZ, are polygenic diseases that are difficult to reproduce in animal models. Substantial progress has been made in studying such pathologies by modeling well-defined genomic lesions that increase the risk of neurodevelopmental diseases. For instance, 22q11.2 deletion syndrome (22q11DS) is a copy number variation disorder that is associated with multiple physical and neuropsychiatric morbidities, such as autism spectrum disorders, attention deficit hyperactivity disorder, anxiety disorder, intellectual disability, Parkinson disease, and others^19–27^, including SCZ^28–34^. Approximately 25% of individuals with 22q11DS are diagnosed with SCZ;^30,33^ in turn, the 22q11.2 deletion is found in one per 100–200 individuals with SCZ^35^, making 22q11DS one of the strongest known genetic risk factors for SCZ^36^.

The 22q11DS is caused by a hemizygous 1.5- to 3-megabase (Mb) microdeletion on chromosome 22. Symptoms are indistinguishable between schizophrenic patients who have the 22q11.2 deletion and those who do not^30,31,37^, suggesting the existence of a common pathogenic mechanism between 22q11DS and SCZ. The 1.5-Mb deletion is thought to be the minimal region containing the genes that account for the increased risk of SCZ among individuals with 22q11DS^38^. Ventricular enlargement is a common neuroanatomical finding in individuals who have a 22q11.2 chromosomal microdeletion. The most frequent changes are seen in the lateral ventricles (LVs) and third ventricle (TV)^13,14,39–42^. The 22q11DS is associated with abnormally dilated LVs^14,39,43–45^. Mouse models of 22q11DS (22q11DS mice) have similar changes in ventricular volume, compared with human 22q11DS carriers^46^. This finding indicates that 22q11DS mice are a valid model of ventricular enlargement. Here we sought to use these mutant mice to elucidate the mechanisms responsible for the increase in ventricular volume in 22q11DS.

Several recent studies have linked abnormalities in 22q11DS mice to the *Dgcr8* (DiGeorge critical region 8) gene^47–51^. *Dgcr8* is important for synthesizing microRNAs (miRNAs), which are negative regulators of mRNA translation^52^. Haploinsufficiency of *Dgcr8* leads to miRNA depletion and abnormally elevated expression of important proteins such as dopamine receptors in various brain regions, thus contributing to SCZ-related phenotypes in 22q11DS mice^47–51^. Here we examined whether *Dgcr8* haploinsufficiency contributes to ventricular enlargement in 22q11DS mice. We also describe a novel pathogenic mechanism involving *Dgcr8*-dependent miRNA depletion in ependymal cells that leads to progressive decelerated motile ciliary beating and age-dependent ventricular enlargement in 22q11DS mouse models.

## Results

### Haploinsufficiency of the 22q11DS gene *Dgcr8* causes age-dependent ventricular enlargement

**Fig. 1:**
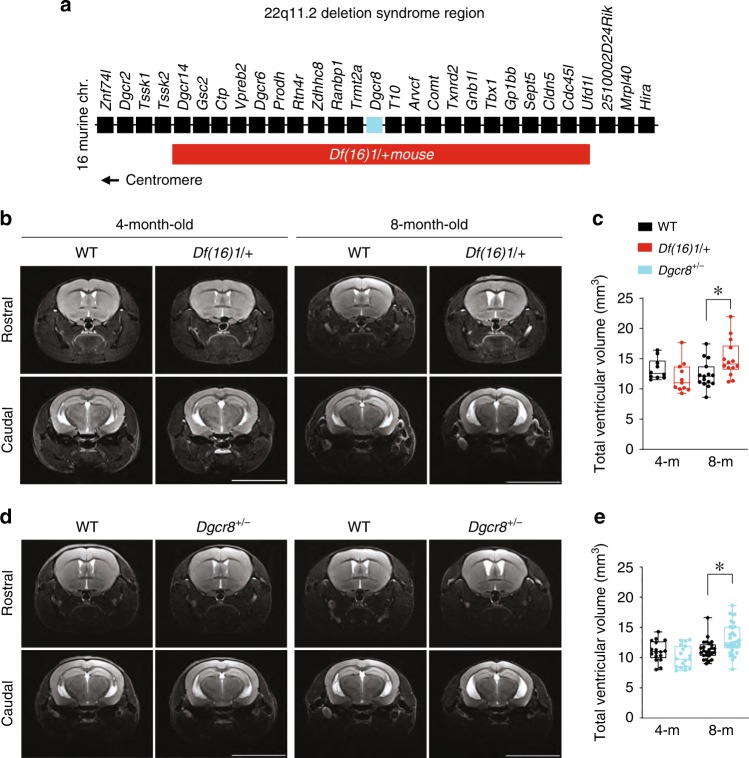
Ventricular enlargement in mouse models of 22q11DS. **a** Map of 22q11DS orthologs deleted in *Df(16)1/+* mice. **b**–**e** Representative MRIs of the rostral and caudal brains (**b**, **d**) and total ventricle volumes (**c**, **e**) of 4- and 8-month-old WT and *Df(16)1/+* mice (**b**, **c**), and WT and *Dgcr8*^*+/−*^ mice (**d**, **e**). Data from the 4-month-old WT (*n* = 10) and *Df(16)/+* (*n* = 11) mice were analyzed using the Mann–Whitney rank-sum test (*U* = 29, *p* = 0.07), and that from 8-month-old WT (*n* = 15) and *Df(16)1/+* (*n* = 14) mice were analyzed in the same manner (*U* = 48, *p < 0.05). Data from the 4-month-old WT (*n* = 17) and *Dgcr8*^*+/−*^ (*n* = 18) mice were analyzed using the two-tailed Student’s *t*-test (*t*_33_ = 1.54; *p* = 0.13), and that from 8-month-old WT (*n* = 26) and *Dgcr8*^*+/−*^ (*n* = 33) mice were analyzed using the Mann–Whitney rank-sum test (*U* = 231, *p *<* 0.01). Scale bars, 8 mm. Data in this figure are graphed as box plots with the median value. Source data are provided as a Source Data file.

We performed magnetic resonance imaging (MRI) analysis in *Df(16)1*/+ murine models of 22q11DS (ref. ^53^; Fig. 1a). The onset of clinical manifestations of psychosis in patients with 22q11DS or SCZ typically occurs during late adolescence or early adulthood (16–30 years)^54,55^. This age is developmentally equivalent to 3–4 months in mice^56^. Thus, we examined the neuroanatomical features in mice that were 4 months or older. We detected a considerable increase in the total ventricular volume in 8-month-old but not 4-month-old *Df(16)1*/+ mice compared to wild-type (WT) littermates (Fig. 1b, c). At the regional level, we observed a significant enlargement of the LVs and TV but no difference in the size of the fourth ventricle or the aqueduct in 8-month-old *Df(16)1*/+ mice compared to age-matched controls (Supplementary Table 1). Similar results were achieved by measuring ventricle-to-brain ratios (VBRs), where ventricular volumes were normalized to the total brain volume (Supplementary Table 1ʹ). Despite the ventricular enlargement, the volumetric characteristics of the total brain, cortex, and hippocampus of *Df(16)1*/+ mice were comparable to those of WT littermates (Supplementary Fig. 1a–e).

*Dgcr8*^*+/−*^ mice showed age-dependent volumetric abnormalities that were similar to those of *Df(16)1*/+ mice. Haploinsufficiency of *Dgcr8* resulted in a significant increase in the ventricular volume in 8-month-old but not 4-month-old mice (Fig. 1d, e). The volume of the LVs and TV increased, but that of the fourth ventricle and aqueduct did not (Supplementary Table 1, 1ʹ). As in *Df(16)1/+* mice, the volumes of the whole brain, cortex, and hippocampus of *Dgcr8*^*+/−*^ did not differ from those of WT littermates at both ages (Supplementary Fig. 1f–j). Although the increase in the ventricular volumes in *Dgcr8*^*+/−*^ mice was similar to that in *Df(16)1/+* mice, it did not replicate it (Supplementary Table 1, 1ʹ). Specifically, the increases in the total ventricular volume and LV volume were not statistically different between 8-month-old *Df(16)1/+* and 8-month-old *Dgcr8*^*+/−*^ mice; however, the increase in the TV was significantly larger in 8-month-old *Df(16)1/+* mice than in 8-month old *Dgcr8*^*+/−*^ mice (Supplementary Table 1, 1ʹ). This suggests that *Dgcr8* haploinsufficiency is a major (but not the sole) contributor to ventricular enlargement in *Df(16)1/+* mice.

To test if deletion of *Dgcr8* limited to the subventricular zone (SVZ), especially in ependymal cells, replicates ventricular enlargement, we generated *Dgcr8* conditional–knockout mice by crossing mice carrying a *Dgcr8* floxed allele^57^ with *Foxj1*^*Cre*^ mice that express *Cre* recombinase in ependymal cells^58^. The *Dgcr8* transcript level in the LV walls was reduced in *Dgcr8*^*+/−*^ mice compared to WT mice (Supplementary Fig. 2b). It was also reduced in *Dgcr8* conditional–knockout mice (Supplementary Fig. 2b). However, only *Foxj1*^*Cre*^;*Dgcr8*^*fl/fl*^ mice but not *Foxj1*^*Cre*^;*Dgcr8*^*fl/+*^ mice showed lower *Dgcr8* transcript levels in the LV walls compared to that in control littermates (Supplementary Fig. 2b). This suggests that conditional heterozygous deletion of *Dgcr8* in ependymal cells does not accurately replicate *Dgcr8* dosage achieved in *Dgcr8*^*+/−*^ mice. Nonetheless, homozygous deletion of *Dgcr8* in ependymal cells has a drastic effect on the ventricular size (Supplementary Fig. 2a, c). The total ventricular volume and volumes of the LVs, TV, and fourth ventricle substantially increased, whereas the aqueduct remained normal in *Foxj1*^*Cre*^;*Dgcr8*^*fl/fl*^ mice (Supplementary Table 2, 2ʹ). Furthermore, the volumes of the whole brain, cortex, and hippocampus were reduced in *Foxj1*^*Cre*^;*Dgcr8*^*fl/fl*^ mice but not in *Foxj1*^*Cre*^;*Dgcr8*^*fl/+*^ mice compared to the control littermates (Supplementary Fig. 2d–h). In addition, SVZ neurogenesis was disrupted in the *Foxj1*^*Cre*^;*Dgcr8*^*fl/fl*^ mice but remained normal in *Foxj1*^*Cre*^;*Dgcr8*^*fl/+*^ mice (Supplementary Fig. 3). Neural progenitor proliferation (measured by the presence of proliferation markers Ki67 and BrdU in the SVZ) (Supplementary Fig. 3a–d), apoptotic cell death (measured by cell positivity for cleaved caspase-3 in the SVZ) (Supplementary Fig. 3e, f), and SVZ neuronal migration (measured by the presence of the neuroblast marker doublecortin and BrdU^+^ cells in the SVZ neuronal migratory stream) (Supplementary Fig. 3g, h) were disrupted in *Foxj1*^*Cre*^;*Dgcr8*^*fl/fl*^ mice but remained normal in *Foxj1*^*Cre*^;*Dgcr8*^*fl/+*^ mice compared to control littermates. These results indicate that *Dgcr8* in the ependymal cells regulates the ventricular volume in a dose-dependent manner. *Foxj1*^*Cre*^;*Dgcr8*^*fl/+*^ mice did not show a decrease in the *Dgcr8* transcript; thus, we could not conclude that *Dgcr8* haploinsufficiency in ependymal cells is necessary for ventricular enlargement. Because 22q11DS is a heterozygous condition, we further focused on investigating mechanisms of age-dependent ventricular enlargement in *Dgcr8*^*+/−*^ mice.

Volumetric changes in *Dgcr8*^*+/−*^ mice were not associated with changes in cerebral spinal fluid (CSF) osmolality (Supplementary Fig. 1k) or abnormal characteristics of neural progenitors in the SVZ (Supplementary Fig. 4). Neural progenitor proliferation (Supplementary Fig. 4a–g), apoptotic cell death (Supplementary Fig. 4h–j), and SVZ neuronal migration (Supplementary Fig. 4k, l) were normal in *Dgcr8*^*+/−*^ mice compared to WT littermates. These observations indicate that although *Dgcr8* haploinsufficiency may cause ventricular enlargement, the mechanism for such a result does not involve changes in CSF composition or SVZ neurogenesis.

### Slower ependymal flow and ciliary beating in the lateral ventricles of *Dgcr8*^*+/−*^ mice

**Fig. 2:**
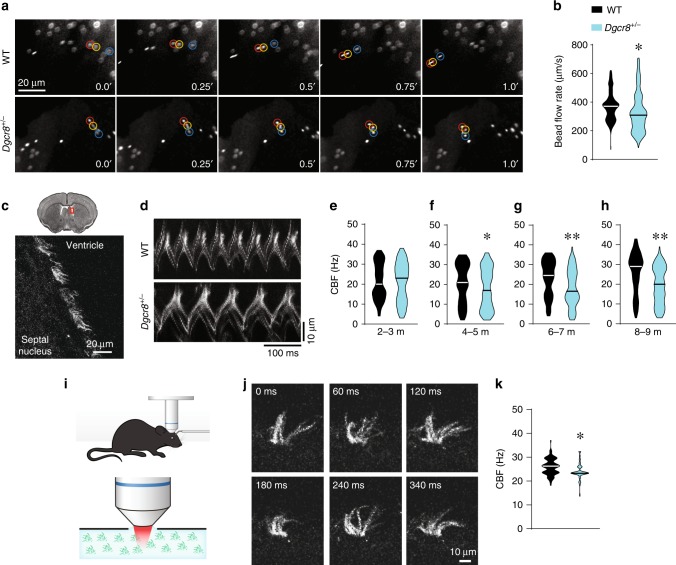
*Dgcr8* haploinsufficiency slows ependymal ciliary beating. **a** Images of fluorescent beads at consecutive time points, which are shown in seconds. **b** The average bead speed is an indicator of the ependymal flow rate in 8-month-old whole mounts from WT (439 beads, 6 mice) and *Dgcr8*^*+/−*^ (450 beads, 6 mice) animals. Data were analyzed using the Mann–Whitney rank-sum test (*U* = 72837, *p *<* 0.001). **c** A coronal section of the brain (top) with the magnified region of the LV wall (bottom) in *Arl13b*^*eGFP*^ mice. Motile cilia were labeled with eGFP and depicted in grayscale mode. **d** Kymographs of cilia beating, which was measured ex vivo brain slices from WT (*Dgcr8*^*+/+*^;*Arl13b*^*eGFP*^) and *Dgcr8*^*+/−*^ (*Dgcr8*^*+/−*^;*Arl13b*^*eGFP*^) mice. **e**–**h** Ciliary beating frequency (CBF) in ex vivo brain slices from WT and *Dgcr8*^*+/−*^ mice at 2–3 months (**e**), 4–5 months (**f**), 6–7 months (**g**), and 8–9 months (**h**) of age. In **e**, 82 ciliary bundles in 3 WT mice and 81 ciliary bundles in 3 *Dgcr8*^*+/−*^ mice (*U* = 3227, *p* = 0.76) were assessed. In **f**, 132 ciliary bundles in 5 WT mice and 143 ciliary bundles in 5 *Dgcr8*^*+/−*^ mice (*U* = 8037, *p *<* 0.05) were assessed. In **g**, 120 ciliary bundles in 4 WT mice and 112 ciliary bundles in 4 *Dgcr8*^*+/−*^ mice (*U* = 4709, **p *<* 0.001) were assessed. In **h**, 145 ciliary bundles in 5 WT mice and 159 ciliary bundles in 5 *Dgcr8*^*+/−*^ mice (*U* = 7116, **p *<* 0.001) were assessed (Mann-Whitney rank-sum test). **i** Experimental schematics for imaging ependymal CBF in vivo in an anesthetized mouse. **j** Representative images of motile cilia in the LV wall of an anesthetized mouse at consecutive time points. **k** Average CBF measured in vivo in WT and *Dgcr8*^*+/−*^ mice (98 ciliary bundles in 3 WT mice, 88 ciliary bundles in 5 *Dgcr8*^*+/−*^ mice; Mann–Whitney rank-sum test; *U* = 2509, *p *<* 0.001). Source data are provided as a Source Data file.

To test whether *Dgcr8* deficiency affects ependymal function, we first examined ependymal flow that is directed by motile ciliary beating. We compared individual fluorescent microbead movements at the apical surface of ependymal cells in acute LV whole-mounts from 8-month-old WT or *Dgcr8*^*+/−*^ mice (Fig. 2a, b, Supplementary Movies 1 and 2). Although the overall direction of ependymal flow was comparable between *Dgcr8*^*+/−*^ and WT mice, the average bead velocity was slower in the mutants (Fig. 2a, b), suggesting that *Dgcr8* haploinsufficiency affects motile ciliary beating. To directly measure motile ciliary beating frequency (CBF), we used linescan fluorescence imaging in brain slices from *Arl13b*^*eGFP*^ transgenic mice, which express green fluorescence protein (GFP) only in primary and motile cilia^59^ (Fig. 2c, d). Using this approach, we observed slower beating of motile cilia in *Dgcr8*^*+/−*^;*Arl13b*^*eGFP*^ mice aged 4–5, 6–7, or 8–9 months but not in those aged 2–3 months, compared to the respective controls (*Dgcr8*^*+/+*^;*Arl13b*^*eGFP*^ littermates) (Fig. 2d–h). Similar results were observed using simultaneous differential interference contrast (DIC) imaging (data not shown). Using these modes of imaging, we observed that in addition to being slower, motile cilia movements were less stereotypical (more asynchronous) in acute brain slices (Supplementary Movies 3 and 4) and whole-mounts (Supplementary Movies 5 and 6) from older (>4 months) *Dgcr8*^*+/−*^ mice compared to WT littermates.

To show ciliary abnormality in vivo, we measured ependymal CBF in anesthetized mice by using two-photon imaging (Fig. 2i). This approach enabled us to detect quickly beating individual cilia within the cilia tuft at the surface of the LV wall (Fig. 2j, Supplementary Movie 7). Similar to results from the ex vivo whole-mount experiments, the ependymal CBF in vivo was slower in 8-month-old *Dgcr8*^*+/−*^;*Arl13b*^*eGFP*^ mice than in *Dgcr8*^*+/+*^;*Arl13b*^*eGFP*^ littermates (Fig. 2k). Together, these results show a progressive reduction in ependymal cilia beating frequency in *Dgcr8*^*+/−*^ mice.

### *Dgcr8* haploinsufficiency does not affect ependymal cell structure or planar polarity

**Fig. 3:**
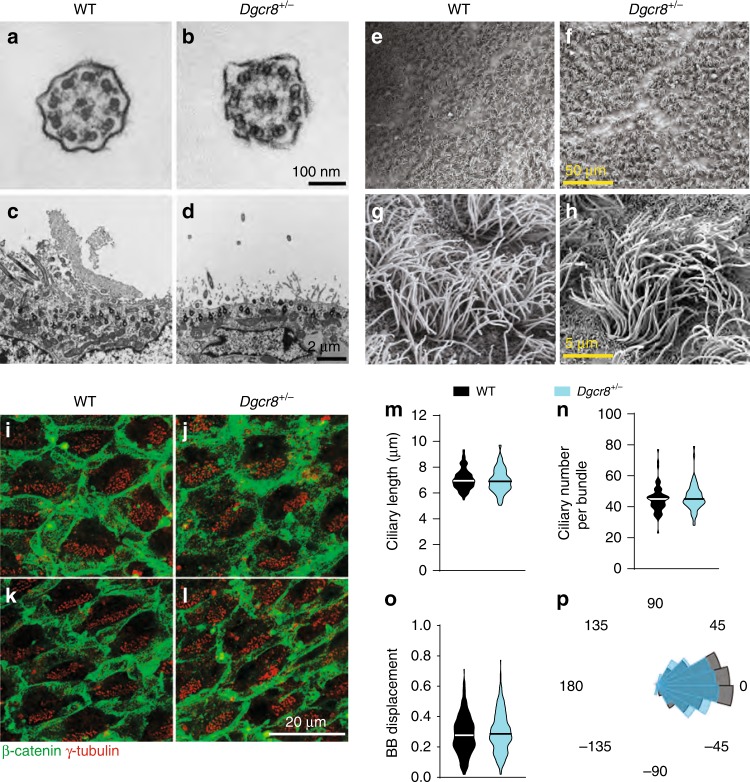
Normal cilia structure and planar polarity of ependymal cells in *Dgcr8*^*+/−*^ mice. **a**–**d** Representative TEM images of motile cilia in ependymal cells of WT and *Dgcr8*^*+/−*^ mice. Cilia are transected at the levels of the axoneme (**a**, **b**) or basal body (**c**, **d**). **e**–**h** Representative SEM images of motile cilia in ependymal cells of WT and *Dgcr8*^*+/−*^ mice. **i**–**l** Confocal images of basal body patch position as an anatomical indicator of ependymal planar polarity in the anterior dorsal (**i**, **j**) and anterior ventral (**k**, **l**) LV walls of WT and *Dgcr8*^*+/−*^ mice. Whole-mount brains were stained with antibodies against β-catenin (green, intercellular junctions) and γ-tubulin (red, basal bodies). **m**–**o** Mean ciliary length (**m**), ciliary number in a bundle (**n**), and distance of the basal body (BB) displacement from the center of an ependymal cell (**o**) in WT and *Dgcr8*^*+/−*^ mice. In **m**, 105 cilia in 3 mice of each genotype were analyzed using the Mann–Whitney rank-sum test (*U* = 5156, *p* = 0.42). In *n*, 72 cilia in three mice of each genotype were analyzed in the same manner (*U* = 2379, *p* = 0.39). In **o**, 309 cells in 3 WT mice and 305 cells in 3 *Dgcr8*^*+/−*^ mice were analyzed using the Shapiro–Wilk normality test (*U* = 44,160, *p* = 0.18). **p** Distribution of the basal body patch is plotted on a polar histogram. Average angles of the individual vectors in each imaged section were normalized to 0°, and distributions of the angles were compared in WT (235 cells, three mice) and *Dgcr8*^*+/−*^ (135 cells, three mice) animals. The data were analyzed using Watson’s U2 two-sample test of homogeneity (*t* = 0.13, *p* = 0.13). Source data are provided as a Source Data file.

The increase in ventricular volume in *Dgcr8*^*+/−*^ mice was not associated with structural changes in ependymal cilia or planar cell polarity. Transmission electron microscopy (TEM) revealed normal microtubule configuration in the 9 + 2 axoneme and basal body docking to the apical membrane of ependymal cells in *Dgcr8*^*+/−*^ mice (Fig. 3a–d). Scanning electron microscopy also showed no abnormalities in morphology, length, or the number of motile cilia on the wall of the LVs in the *Dgcr8*^*+/−*^ mice compared to WT littermates (Fig. 3e–h, m, n). Furthermore, planar polarity of ependymal cells was not altered in *Dgcr8*^*+/−*^ mice (Fig. 3i–l). Planar polarity within the ependymal cells is determined by the location of the microtubule-based basal bodies, which give rise to the motile cilia^60^. Co-labeling of the cell adherens junction and basal body with antibodies against β-catenin and γ-tubulin in the LV wall showed preserved cell polarity in both genotypes. Displacement of the basal body patch from the cell center, an indicator of translational polarity, was similar between the genotypes (Fig. 3o). Furthermore, the alignment of rotational orientation of basal bodies, which is thought to determine the direction of motile cilia beating^61–66^, was not altered in *Dgcr8*^*+/−*^ mice (Fig. 3p).

### Dysregulation of the dopamine receptor Drd1 in ependymal cells of *Dgcr8*^*+/−*^ mice

**Fig. 4:**
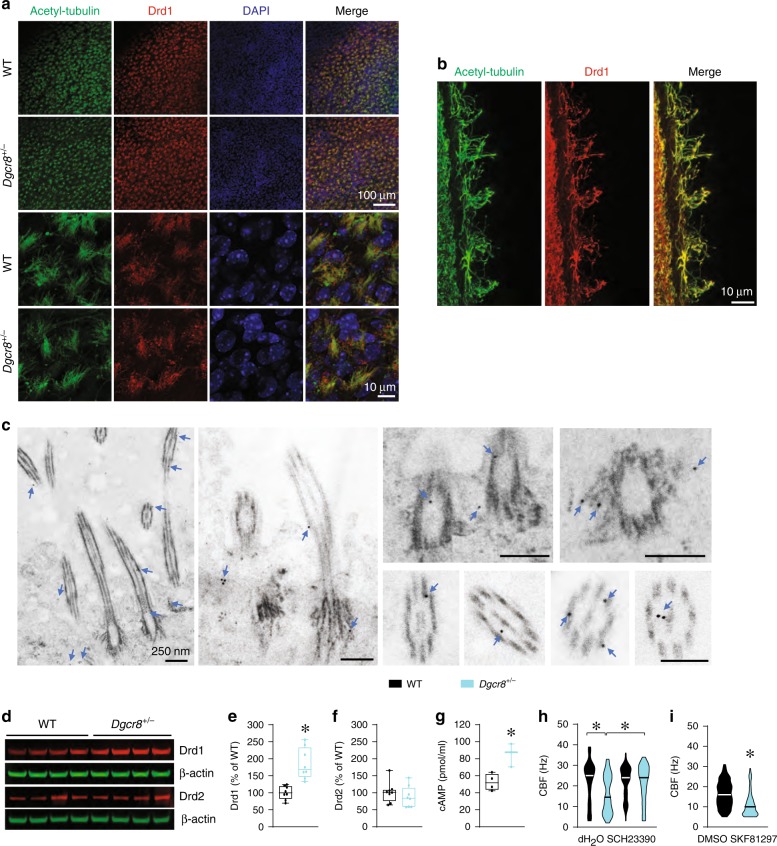
*Dgcr8* haploinsufficiency increases the expression of dopamine receptor Drd1 in the LV wall. **a** Confocal images of Drd1 expression in the ependymal cells of whole-mount LV walls from WT or *Dgcr8*^*+/−*^ mice. Acetyl-tubulin antibody is used as a marker of motile cilia and DAPI stains the nuclei. **b** Super-resolution microscopic images of coronal brain sections from the WT (*Foxj1*^*Cre*^*;Drd1*^*+/+*^) mice show expression of Drd1 in motile cilia. Acetyl-tubulin antibody is used as a marker of motile cilia. **c** Immunogold EM images show localization of immunogold-tagged Drd1 in the cytoplasm of ependymal cells and near the microtubules of cilia, including the axoneme, basal body, and distal appendages of the basal body (arrows). **d** Representative immunoblotting of Drd1 and Drd2 in the LV wall of WT and *Dgcr8*^*+/–*^ mice. **e**–**g** Quantification of Drd1 (**e**) and Drd2 (**f**) protein levels and the cAMP level (**g**) in the LV wall of WT and *Dgcr8*^*+/–*^ mice. In **e**, the data represent eight mice per group and were analyzed using the two-tailed Student’s *t*-test (*t*_14_ = 4.80, *p *<* 0.001). In **f**, eight mice per group were included, and the data were analyzed in the same manner (*t*_14_ = 0.89, *p* = 0.39). In **g**, 4 WT and 3 *Dgcr8*^*+/−*^ mice were included in each experiment, which were run in triplicate and analyzed using the two-tailed Student’s *t*-test (*t*_5_ = 3.72, *p *<* 0.05). **h**, **i** Average CBF in ex vivo brain slices from 6-month-old WT and *Dgcr8*^*+/−*^ mice in the absence (85 ciliary bundles in 3 WT mice and 88 ciliary bundles in 3 *Dgcr8*^*+/−*^ mice) or presence of Drd1 antagonist SCH23390 (93 ciliary bundles in 3 WT mice and 85 ciliary bundles in 3 *Dgcr8*^*+/−*^ mice) (**h**). Data were analyzed using one-way ANOVA (*F*_3_ = 24.99, *p *<* 0.001) (**h**) and in slices from 4- to 5-month-old WT mice in the presence of vehicle (DMSO) (67 ciliary bundles in 3 mice) or the Drd1 agonist SKF81297 (60 ciliary bundles in 3 mice) **i**. Data were analyzed using the Mann-Whitney rank-sum test (*U* = 1043, *p *<* 0.001) (**i**). Source data are provided as a Source Data file.

Previous reports indicate that Dgcr8 regulates the expression of dopamine receptors through specific miRNAs^48^. Dopamine receptors are present not only on neurons but also on ciliated ependymal cells^67,68^, where catecholamine projections from the subependymal layer may form synapses with them^67,69^. First, we determined whether Drd1 is present in the motile cilia. Immunostaining of the LV wall for motile cilia markers (i.e., acetylated tubulin or Arl13b) and dopamine receptors revealed that Drd1s localize to the motile cilia (Fig. 4a, Supplementary Fig. 5a, b) in both WT and *Dgcr8*^*+/−*^ mice (Fig. 4a). Immunolabelling and super-resolution imaging confirmed the presence of Drd1s in the motile cilia (Fig. 4b) and revealed that immunolabeling of the motile cilia with antibodies against Drd1 was specific (Supplementary Fig. 5c). To further confirm the subcellular localization of Drd1 in ependymal cells, we performed immunogold EM experiments in sections containing ependymal cells in WT mice. The gold particles were detected in the cytoplasm of ependymal cells and cilia, including in the axoneme, near the outer and central microtubules, and at the basal body and its distal appendages (Fig. 4c). In contrast, Drd2s were not detected in the motile cilia (Supplementary Fig. 5d). Together, these experiments determined that Drd1 receptors are present in the motile cilia.

To determine if *Dgcr8* haploinsufficiency affects dopamine receptor expression, we performed Western blotting for Drd1 and Drd2 proteins in extracts from the LV walls. (Note, the LV wall contains several cell types, including ependymal cells.) The expression level of Drd1 but not Drd2 was significantly elevated in *Dgcr8*^*+/−*^ mice compared to WT controls (Fig. 4d–f). Consistent with this finding, the levels of intracellular cAMP, the downstream effector of Drd1 signaling, was also significantly increased in the LV wall of *Dgcr8*^*+/−*^ mice (Fig. 4g). Furthermore, blocking Drd1 activity with SCH23390, a Drd1-specific antagonist, rescued abnormal ependymal function in *Dgcr8*^*+/−*^ mice (Fig. 4h). SCH23390 significantly accelerated ependymal CBF (*p* *<* 0.001), compared to vehicle in slices from *Dgcr8*^*+/−*^ mice, making their CBF comparable to that of WT mice. Conversely, activation of Drd1 by SKF81297, a Drd1-specific agonist, mimicked the *Dgcr8*^*+/−*^ ciliary beating phenotype in WT mice (Fig. 4i). Application of SKF81297 slowed ependymal CBF compared to vehicle treatment in slices from WT mice. Together, these results suggest that *Dgcr8* haploinsufficiency upregulates Drd1 in the LV walls, and this reduces ependymal CBF. Because a major function of Dgcr8 is production of miRNAs, we next examined whether a Dgcr8–miRNA–Drd1 mechanism contributes to the deceleration of ependymal CBF in 22q11DS mice.

### Depleting miR-382-3p or miR-674-3p or deleting their seed sites on *Drd1* 3ʹUTR decelerates ependymal ciliary beating and mediates ventricular enlargement

**Fig. 5:**
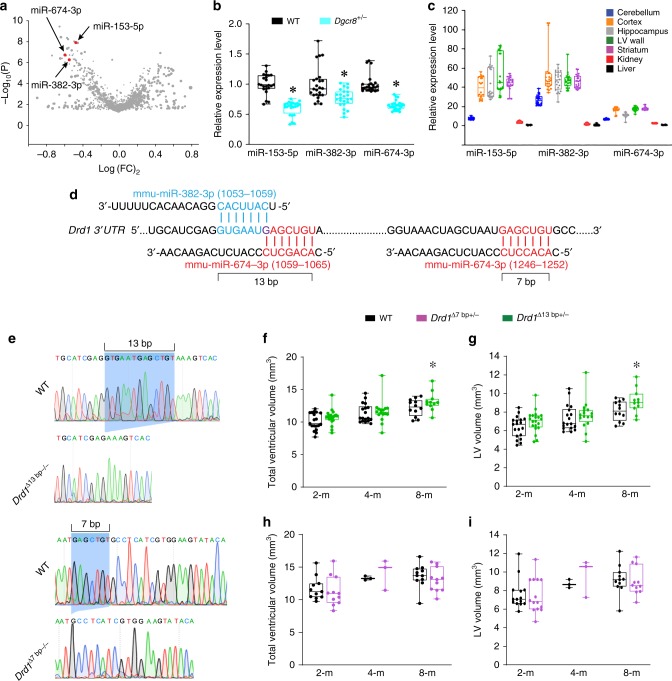
*Drd1*-targeting miRNAs are depleted in *Dgcr8*^*+/−*^ mice, and deletion of miRNA seed site on *Drd1* 3ʹUTR causes ventricular enlargement. **a** A miRNA microarray volcano plot depicting changes in miRNA expression in the LV wall of male *Dgcr8*^*+/−*^ mice compared to that in male WT mice. **b** Normalized relative levels of miR-153-5p, miR-382-3p, and miR-674-3p measured by qPCR in the LV wall in WT (8 mice) and *Dgcr8*^*+/−*^ littermates (8 mice). The data were analyzed using Mann–Whitney rank-sum test (*U* = 12, *p *<* 0.001 for miR-153-5p; *U* = 133, *p *<* 0.001 for miR-382-3p; *U* = 0, *p *<* 0.001 for miR-674-3p). **c** Normalized relative expression of miR-153-5p (8 mice), miR-382-3p (8 mice), and miR-674-3p (8 mice) in cerebellum, cortex, hippocampus, LV wall, striatum, kidney, and liver, as measured by qPCR. Data were analyzed using Kruskal–Wallis one-way analysis (*H*_6_ = 133.96, *p* *<* 0.001 for miR-153-5p; *H*_6_ = 127.59, *p* *<* 0.001 for miR-382-3p; and *H*_6_ = 149.63, *p* *<* 0.001 for miR-674-3p). **d** The predicted miRNA target sites of miR-382-3p (blue) and miR-674-3p (red) on the *Drd1* 3ʹUTR (NM_010076). The overlapped nucleotide *G* between the two miRNA seed sites within the 13-bp sequence is indicated in purple. **e** Validation of the 13-bp and 7-bp deletions in the *Drd1* 3ʹUTR of the *Drd1*^*Δ13bp−/−*^ and *Drd1*^*Δ7bp−/−*^ mice by cDNA sequencing. The blue shaded areas indicate the 13- and 7-bp seed sites in the WT *Drd1* 3ʹUTR, which is deleted in *Drd1*^*Δ13bp−/−*^ and *Drd1*^*Δ7bp−/−*^ mice, respectively. **f**–**i** Total ventricle volumes (**f**, **h**) and LV volumes (**g**, **i**) of 2-month-old (*n* = 19/20 for WT and *Drd1*^*Δ13bp+/−*^ mice; *n* = 11/11 for WT and *Drd1*^*Δ7bp+/−*^ mice), 4-month-old (*n* = 18/16 for WT and *Drd1*^*Δ13bp+/−*^ mice; *n* = 3/3 for WT and *Drd1*^*Δ7bp+/−*^ mice), and 8-month-old (*n* = 12/11 for WT and *Drd1*^*Δ13bp+/−*^ mice; *n* = 11/11 for WT and *Drd1*^*Δ7bp+/−*^ mice) mice. Data were analyzed using two-way ANOVA. **f**, **h** Age: *F*(2,90) = 18.81, *p* *<* 0.001, genotype: *F*(1,90) = 4.078, *p = 0.046 (f); Age: *F*(2,44) = 6.5, *p* *<* 0.003, genotype: *F*(1,44) = 0.017, *p* = 0.896 (h). **g**, **i** Age: *F*(2,90) = 17.12, *p* *<* 0.001, genotype: *F*(1,90) = 6.06, *p = 0.016 (g); Age: *F*(2,44) = 5.68, *p* *<* 0.006, genotype: *F*(2,44) = 0.03, *p* = 0.861 (**i**). Source data are provided as a Source Data file.

To identify the miRNAs involved in the Dgcr8–miRNA–Drd1 mechanism, we performed miRNA microarray analysis in the LV wall of 8-month-old *Dgcr8*^*+/−*^ and WT littermates. Among the miRNAs that potentially target the 3ʹ untranslated region (UTR) of the *Drd1* transcript (on the basis of the miRWalk and TargetScan miRNA-target-prediction algorithms), only three miRNAs (miR-153-5p, miR-382-3p, and miR-674-3p) were significantly decreased in *Dgcr8*^*+/−*^ mice (Fig. 5a, Supplementary Table 3). The RT-qPCR analysis verified that the levels of all three *Drd1*-targeting miRNAs were significantly reduced in *Dgcr8*^*+/−*^ mice compared to WT mice (Fig. 5b). All three miRNAs were substantially enriched in the brain, including the LV wall, compared to non-brain areas such as kidney or liver (Fig. 5c). Of the three identified miRNAs, overexpression of two, miR-382-3p (miR-382-3p OE) and miR-674-3p (miR-674-3p OE), decreased the levels of *Drd1* transcripts in vitro (Supplementary Fig. 6). Therefore, in further experiments we focused on these two miRNAs as the mediators of the Dgcr8–miRNA–Drd1 mechanism. The miR-382-3p is conserved across species, including humans and mice, but miR-674-3p does not have a human homolog. Interestingly, the predicted miRNA-target sites of miR-382-3p and miR-674-3p overlapped on the *Drd1* 3ʹUTR (Fig. 5d). The 13-bp sequence containing seed sequences from both miRNAs is unique throughout all 3ʹUTRs of reference-sequence genes in the mouse genome (GRCm38/mm10 assembly). The miR-674-3p has an additional 7-bp binding site in the *Drd1* 3ʹUTR (Fig. 5d). To test whether the miR-382-3p– or miR-674-3p–binding sites are involved in ventricular enlargement, we generated mice with deletions of the 13-bp seed site (Drd1^Δ13bp^) and the 7-bp seed site (Drd1^Δ7bp^), respectively, in the *Drd1* 3ʹUTR by using the CRISPR/Cas9 approach (Fig. 5e). Both *Drd1*^*Δ7bp+/−*^ and *Drd1*^*Δ13bp+/−*^ mice developed normally and had no detectible gross abnormalities, compared to their respective WT controls (data not shown). *Drd1*^*Δ13bp+/−*^ mice showed age-dependent and region-specific ventricular enlargement (Fig. 5f, g). Specifically, 8-month-old but not younger *Drd1*^*Δ13bp+/−*^ mice had significantly enlarged total and LV volumes compared to WT littermates (Fig. 5f, g, Supplementary Table 4, 4ʹ). In contrast, we did not detect any differences in ventricular volumes in *Drd1*^*Δ7bp+/−*^ mice compared to control mice (Fig. 5h, i). This suggests that the unique 13-bp miR-382-3p/miR-674-3p *Drd1* seed site but not the 7-bp miR-674-3p *Drd1* seed site mediates the effect of these miRNAs in the ventricular-enlargement phenotype.

**Fig. 6:**
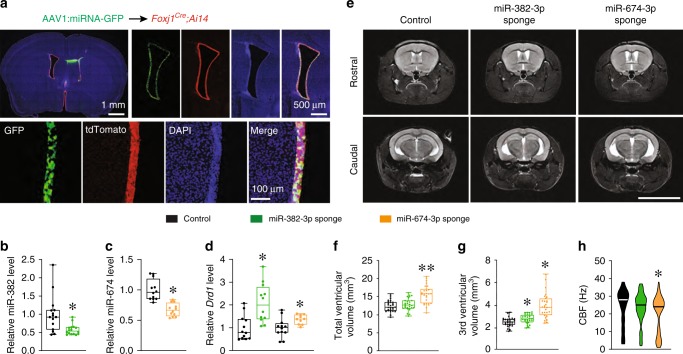
Knockdown of *Drd1*-targeting miRNAs in the ependymal cells mimics ventricular enlargement seen in 22q11DS mice. **a** Confocal images of miRNA (GFP) expression in the coronal sections of *Foxj1*^*Cre*^;*Ai14* brains. Cre expression is marked with tdTomato, and DAPI counterstains the nuclei. **b**–**d** Normalized levels of miR-382-3p (**b**), miR-674-3p (**c**), and *Drd1* mRNA (**d**) in the LV wall injected with AAV1 expressing GFP or miR-382-3p sponge (**b**, **d**) or miR-674-3p sponge (**c**, **d**). In **b**, three mice per group were analyzed using the Mann–Whitney rank-sum test (*U* = 42, *p *<* 0.01). In **c**, four mice per group were analyzed using the two-tailed Student’s *t*-test (*t*_22_ = 5.60, *p *<* 0.001). In **d**, the data were analyzed using the two-tailed Student’s *t*-test (*t*_22_ = 3.71, *p *<* 0.001 for miR-382-3p sponge; *t*_20_ = 2.47, *p *<* 0.05 for miR-674-3p sponge). **e** Representative MRIs of the rostral and caudal brains injected with an AAV1 expressing a scramble control, miR-382-3p sponge, or miR-674-3p sponge. **f**–**h** The total ventricular volume (**f**), third ventricular volume (**g**), and CBF (**h**) of 8-month-old WT mice injected with AAV1 expressing scramble (control, 27 mice), miR-382-3p sponge (28 mice), or miR-674-3p sponge (28 mice) into the intracerebroventricular space at 2–3 months of age. Data were analyzed using one-way ANOVA, **f**: *F*_3_ = 21.82, **p *<* 0.001, **g**: *H*_2_ = 35.98, *p *<* 0.001. **h** control (136 ciliary bundles in four mice), miR-382-3p sponge (124 ciliary bundles in four mice), and miR-674-3p sponge (120 ciliary bundles in five mice, *H*_2_ = 15.69, *p *<* 0.001). Scale bar, 8 mm. Source data are provided as a Source Data file.

To test if depletion of miR-382-3p or miR-674-3p mimics the cilia deceleration and ventricular enlargement in WT mice in vivo, we generated sponges specific for miR-382-3p or miR-674-3p. The miR-382-3p or miR-674-3p effectively inhibited reporters containing the sponge sequence in vitro, as measured by the luciferase assay (Supplementary Fig. 7). To deliver these sponges in vivo, we generated the recombinant adeno-associated virus serotype 1 (AAV1) encoding GFP under the ubiquitous CAG promoter (AAV1-CAG-GFP). We injected AAV1-CAG-GFP intracerebroventricularly in *Foxj1*^*Cre*^*;Ai14* mice, which express tdTomato in ependymal cells. Approximately 3 weeks after injection, we detected robust co-labeling of GFP with tdTomato, indicating that the AAV1 efficiently and specifically infected the ependymal cells (Fig. 6a and Supplementary Fig. 8). The GFP signal was co-localized with tdTomato^+^ and S100β^+^ ependymal cells but not with neural progenitor markers GFAP and Ki67 (Supplementary Fig. 8b), indicating that ependymal cells have a strong affinity for AAV1, as previously shown^70^. In contrast, AAV5 viruses were less specific and infected ependymal cells and other cells, including neurons in other brain areas (data not shown).

To use this AAV1 tropism for specific molecular manipulations in ependymal cells, we constructed an AAV1 containing miR-382-3p, miR-674-3p sponges, or scramble control (AAV1–CAG–GFP–miR-382-3p sponge, AAV1–CAG–GFP–miR-674-3p sponge, and AAV1–CAG–GFP–scramble). The infection with AAV1 encoding the miR-382-3p or miR-674-3p sponge significantly reduced the levels of respective miRNAs and increased the expression of the *Drd1* transcript in the LV wall of WT mice (Fig. 6b–d). The miR-674-3p sponge expression in ependymal cells of WT mice resulted in ventricular enlargement, including increased volume of the TV (Fig. 6e–g, Supplementary Table 5, 5ʹ). The miR-382-3p sponge increased the volume of the TV but did not affect the total ventricular volume (Fig. 6g, Supplementary Table 5, 5ʹ). These significant differences in ventricle size were not accompanied by other aberrant neuroanatomical features or changes in ependymal planar polarity (Supplementary Fig. 9). Furthermore, the AAV1 expressing the miR-674-3p sponge significantly reduced the ependymal CBF compared to control virus in WT mice (Fig. 6h). Ependymal CBF also appeared to be reduced when ependymal cells were infected with AAV1 expressing the miR-382-3p sponge, but that reduction was not significant. Together, these results suggest that depletion of miR-382-3p or miR-674-3p, which occurs in *Dgcr8*^*+/−*^ mice, slows ependymal CBF and induces ventricular enlargement.

### Replenishing miR-382-3p or miR-674-3p rescues the cilia-beating deficit and ventricular enlargement in 22q11DS mice

Given that depletion of miR-382-3p or miR-674-3p leads to ventricular enlargement in 22q11DS mice, we sought to rescue this phenotype by overexpressing these miRNAs in the ependymal cells. To this end, we constructed AAV1 expressing miR-382-3p or miR-674-3p with GFP (AAV1–miR-382-3p OE and AAV1–miR-674-3p OE). After we injected AAV1–miR-382-3p OE or AAV1–miR-674-3p OE intracerebroventricularly, the expression of each miRNA was increased, compared to the levels in mice that received injections with a control virus (Supplementary Fig. 10a, b). This coincided with reduced *Drd1* mRNA levels in the LV wall of *Df(16)1/+* mice (Supplementary Fig. 10c). Neither AAV1–miR-382-3p OE nor AAV1–miR-674-3p OE affected *Drd1* mRNA levels in WT mice. However, overexpression of AAV1–miR-674-3p significantly reduced *Drd1* mRNA levels in *Df(16)1/+* mice (*p* < 0.001), which became indistinguishable from WT. *Drd1* mRNA levels in *Df(16)1/+* mice were also significantly reduced (*p* < 0.001) after injection of AAV1–miR-382-3p OE but still remained higher than in WT mice (Supplementary Fig. 10c).

**Fig. 7:**
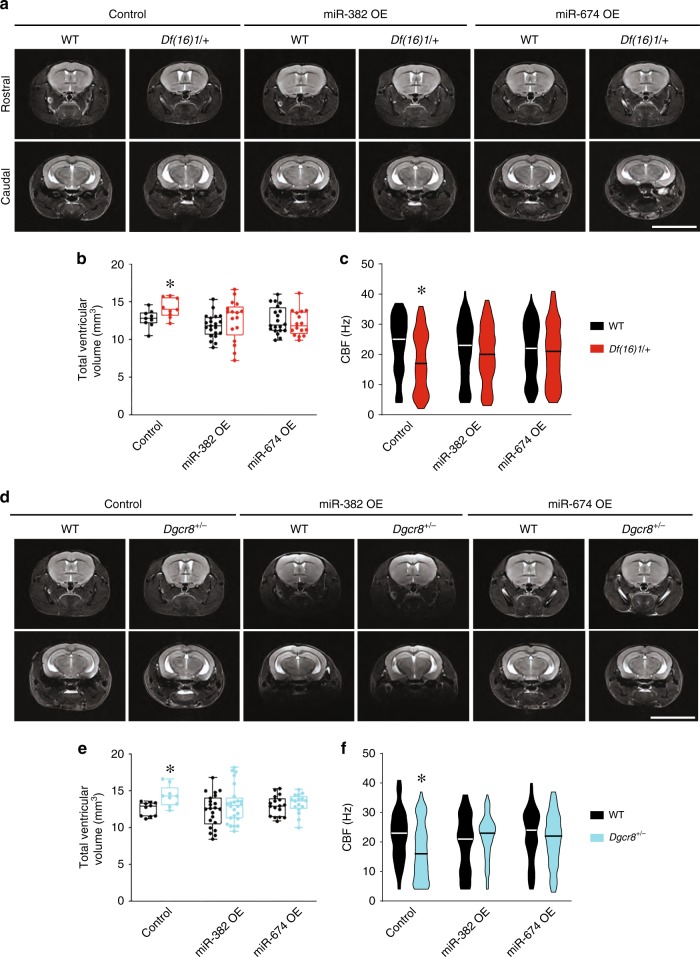
Overexpression of *Drd1*-targeting miRNAs in the ependymal cells rescues 22q11DS-mediated ventricular enlargement. **a** Representative MRIs of the brains of WT or *Df(16)+* mice injected with an AAV1 expressing scramble control, miR-382-3p (miR-382 OE), or miR-674-3p (miR-674 OE). **b** Mean total ventricular volume of 8-month-old WT and *Df(16)/+* mice injected with an AAV1 expressing scramble control (control: 9 WT mice, 9 *Df(16)/+* mice; two-tailed Student’s *t*-test; *t*_16_ = 2.39, *p *<* 0.05), miR-382-3p (miR-382 OE: 21 WT mice, 17 *Df(16)/+* mice; two-tailed Student’s *t*-test; *t*_36_ = 1.14, *p* = 0.26), or miR-674-3p (miR-674 OE: 20 WT mice, 16 *Df(16)/+* mice; two-tailed Student’s *t*-test; *t*_34_ = 0.66, *p* = 0.52) into the intracerebroventricular space at 2–3 months of age. **c** CBF measured in ex vivo brain slices from 8-month-old WT mice injected with an AAV1 expressing scramble control (control: 111 ciliary bundles in 5 WT mice, 90 ciliary bundles in 5 *Df(16)/+* mice; Mann–Whitney rank-sum test; *U* = 3497, *p *<* 0.001), miR-382-3p (miR-382 OE: 139 ciliary bundles in 5 WT mice, 156 ciliary bundles in 5 *Df(16)/+* mice; Mann-Whitney rank-sum test; *U* = 9962, *p* = 0.23), or miR-674-3p (miR-674 OE: 151 ciliary bundles in 5 WT mice, 148 ciliary bundles in 5 *Df(16)/+* mice; Mann–Whitney rank-sum test; *U* = 10,622, *p* = 0.46. **d** Representative MRIs of the brains of WT or *Dgcr8*^+/−^ mice injected with an AAV1 expressing scramble (Control), miR-382-3p (miR-382 OE), or miR-674-3p (miR-674 OE). **e** Mean total ventricular volume of 8-month-old WT or *Dgcr8*^*+/−*^ mice injected with AAV1 expressing scramble control (Control: 10 WT mice, 9 *Dgcr8*^*+/−*^ mice; two-tailed Student’s *t*-test; *t*_17_ = 3.20, *p *<* 0.01), miR-382-3p (miR-382 OE: 22 WT mice, 24 *Dgcr8*^*+/−*^ mice; two-tailed Student’s *t*-test; *t*_44_ = 1.17, *p* = 0.25), or miR-674-3p (miR-674 OE: 17 WT mice, 16 *Dgcr8*^*+/−*^ mice; two-tailed Student’s *t*-test; *t*_31_ = 0.58, *p* = 0.57). **f** CBF measured in ex vivo brain slices from 8-month-old WT mice injected with AAV1 expressing scramble control (Control: 153 ciliary bundles in 5 WT mice, 128 ciliary bundles in 5 *Dgcr8*^*+/−*^ mice; Mann-Whitney rank-sum test; *U* = 6736.5, *p *<* 0.001), miR-382-3p (miR-382 OE: 134 ciliary bundles in 5 WT mice, 131 ciliary bundles in 4 *Dgcr8*^*+/−*^ mice; Mann-Whitney rank-sum test; *U* = 7640.5, *p* = 0.07), or miR-674-3p (miR-674 OE: 162 ciliary bundles in 6 WT mice, 168 ciliary bundles in 6 *Dgcr8*^*+/−*^ mice; Mann–Whitney rank-sum test; *U* = 12,165, *p* = 0.10). Scale bars, 8 mm. Source data are provided as a Source Data file.

AAV1–miR-382-3p OE and AAV1–miR-674-3p OE but not a control AAV1 injected intracerebroventricularly into 2- to 3-month-old *Df(16)1/+* mice prevented ventricular enlargement measured at 8 months of age (Fig. 7a, b, Supplementary Table 6, 6ʹ). The total ventricular volume was substantially larger in *Df(16)1/+* mutants than in WT mice when both genotypes were injected with the control virus. However, this ventricular enlargement was eliminated when WT and *Df(16)1/+* mice were injected with AAV1–miR-382-3p OE or AAV1–miR-674-3p OE (Fig. 7b). AAV1–miR-382-3p OE and AAV1–miR-674-3p OE did not affect ventricular volumes in WT mice, but ventricular volume was significantly smaller in *Df(16)1/+* mutants injected with AAV1–miR-674-3p OE than those injected with control AAV1 (*p* = 0.006). A trend of decreased total ventricular volume was also observed in *Df(16)1/+* mice injected with AAV1–miR-382-3p OE, but that decrease was not significant (*p* = 0.055) (Supplementary Table 6, 6ʹ).

The normalization of the ventricular size in *Df(16)1/+* mice injected with AAV1–miR-674-3p OE or AAV1–miR-382-3p OE was accompanied by the rescue of ciliary beating (Fig. 7c). Ependymal CBF was significantly lower in *Df(16)1/+* mice than WT littermates when both genotypes were injected with the control virus. However, this difference was eliminated when mice were injected with either AAV1–miR-674-3p OE or AAV1–miR-382-3p OE. Similar to the ventricular volume, ependymal CBF in WT mice was not affected by AAV1–miR-674-3p OE or AAV1–miR-382-3p OE. However, AAV1–miR-674-3p OE significantly increased ependymal CBF in *Df(16)1/+* mice (*p* = 0.04). The CBF in *Df(16)1/+* mice injected with AAV1–miR-382-3p OE also appeared to be faster than that in mice injected with control virus, but the increase was not significant (*p* = 0.08).

The normalization of the ventricular volume and CBF following intracerebroventricular injections of AAV1–miR-674-3p OE or AAV1–miR-382-3p OE was also observed in *Dgcr8*^*+/−*^ mice (Fig. 7d–f). *Dgcr8*^*+/−*^ mutants injected with the control virus had significantly enlarged ventricles and decreased CBF. However, injection of AAV1–miR-674-3p OE or AAV1–miR-382-3p OE eliminated these differences between WT and *Dgcr8*^*+/−*^ littermates. In both measures, neither virus affected WT mice but rescued the defects in *Dgcr8*^*+/−*^ mutants. AAV1–miR-674-3p OE significantly decreased the ventricular volume (*p* = 0.04) and increased ependymal CBF (*p* *<* 0.01) in *Dgcr8*^*+/−*^ mice. AAV1–miR-382-3p OE appeared to decrease ventricular volume, but the change was not significant (*p* = 0.08); however, it significantly increased CBF (*p* *<* 0.001) in *Dgcr8*^*+/−*^ mice (Fig. 7e, f).

Together, these results indicate that *Dgcr8* haploinsufficiency is a genetic factor significantly contributing to progressive enlargement of the ventricles in 22q11DS mice. Both ventricular enlargement and the deficit in ciliary beating are mediated by the Dgcr8–miR-382-3p/miR-674-3p–Drd1 mechanism.

## Discussion

Here we identified a novel mechanism of age-dependent ventricular enlargement in mouse models of 22q11DS. We have shown that haploinsufficiency of the 22q11DS gene *Dgcr8*, which is involved in miRNA biosynthesis, leads to depletion of miR-382-3p and miR-674-3p and elevation of Drd1 expression in the LV wall. We also determined that in ependymal cells Drd1 is expressed on the motile cilia. The increased expression of Drd1 reduces ependymal CBF, which is associated with age-dependent ventricular enlargement in 22q11DS mice. Overall, these experiments suggest that *Dgcr8* haploinsufficiency operating via the Dgcr8–miR-382-3p/miR-674-3p–Drd1 mechanism is a major contributing factor to ventricular enlargement in 22q11DS mice. However, our data do not rule out that other genes within the microdeletion may also contribute to age-dependent ventricular enlargement in 22q11DS.

Multiple factors may contribute to ventricular enlargement, including stenosis of the aqueduct of Sylvius, impaired CSF homeostasis, aging, certain diseases, and cilia dysfunction^71,72^. Aqueduct stenosis and abnormal production or adsorption of CSF can cause the most drastic changes in the ventricular volume, leading to hydrocephalus, especially during early development. There was no stenosis of the aqueduct in 22q11DS mouse brains, indicating that this enlargement of ventricles was nonobstructive. CSF osmolality was also not changed in the mutant brains. The slow developmental trajectory of ventricular enlargement in 22q11DS mice starkly contrasts with that of rapidly developing congenital childhood hydrocephalus, a severe neurological disorder with high mortality^73^. It also contrasts with occasional sporadic hydrocephalus in mice, which is much more severe and evident at birth. Hydrocephalic animals develop severe lesions and die at 20–40 days of age^73^, but there is no abnormal mortality in 22q11DS mice at this age. In humans and mice, ventricular volumes progressively increase during normal aging;^6,7,74^ this enlargement also has been often observed in neurological disorders, such as Alzheimer disease, vascular dementia, bipolar disorder, copy number variation disorders, and Parkinson disease^1–5,75^. However, evidence of ventricular enlargement in SCZ is overwhelming and reported in more than 80% of SCZ studies^13^. The slowly progressing ventricular enlargement described here resembled that which occurs in SCZ patients^14,76,77^. The late onset of ventricular enlargement in 22q11DS mice also resembles idiopathic normal-pressure hydrocephalus. This normally occurs in older patients and is frequently misdiagnosed with co-morbidities (i.e., Parkinson disease), and can be improved by CSF drainage^78–80^. Similar clinical management could be potentially used in 22q11DS patients with advanced ventricular enlargement.

Structural neuroimaging studies of children with 22q11DS have reported multiple volumetric brain abnormalities^43,44,81^. A recent study indicated the existence of three subgroups within 22q11DS based on severity of changes in midline structures^82^. The changes we observed in 22q11DS mouse models and *Dgcr8*^*+/−*^ mice were relatively mild and correspond to subgroup 1 or 2 of this classification. Indeed, we observed no difference in total brain size, total (brain–CSF) volumes, cortical thickness, or hippocampal volume in these mutant mice compared to WT controls. The only consistent change we observed in 22q11DS and *Dgcr8*^*+/−*^ mice was an age-dependent enlargement of the LV and TV volumes.

The cumulative data show a strong association between aberrant ciliogenesis or ciliary beating and ventricular enlargement^72,83–87^. In the present study, we detected a defect in cilia beating that was associated with ventricular enlargement in 22q11DS mice. Slower motile cilia beating in 22q11DS mice may arise from structural or functional abnormalities. EM and light imaging revealed that 22q11DS mice have no structural defects in the motile cilium. Planar polarity in ependymal cells was also normal in *Dgcr8*^*+/−*^ mice. The unidirectional flow of CSF is generated by the well-coordinated stroke of motile cilia and is thought to depend on the planar polarity of the cells^61–66^. Furthermore, we detected no abnormalities in SVZ neurogenesis (i.e., cell proliferation, apoptosis, or neuronal migration into the olfactory bulb) in mice with heterozygous deletion of *Dgcr8*. This is consistent with the notion that heterozygous *Dgcr8* deletion has a negligible effect on SVZ neurogenesis, whereas homozygous *Dgcr8* deletion substantially affects SVZ neurogenesis. One report indicates that heterozygous deletion of *Dgcr8* does not affect proliferation of adult neural stem cells in the SVZ (but reduces cell proliferation and neurogenesis in the adult hippocampus)^88^. Another report indicates that mitotic frequency of basal progenitors in the late (E16.5) but not early (E13.5) stage of embryonic SVZ development is slightly reduced in the *Dgcr8*^*+/−*^ mice^89^. When corticogenesis was investigated in mice with conditional deletion of *Dgcr8* using *Emx1*^*Cre*^, there was a substantial defect in homozygous mice but not in heterozygous mice, in terms of the cortical or SVZ thickness at E13.5. The homozygous ablation of *Dgcr8* and, to a much less extent, heterozygous ablation of *Dgcr8* affected the maintenance and differentiation of apical and basal progenitors in the cortical VZ/SVZ and reduced the expression of Ctip2 and Sox5 in newborn neurons during early corticogeneisis^90^. Our data show that homozygous deletion of *Dgcr8* in Foxj1^+^ ependymal cells leads to ventricular enlargement but through a severe defect in SVZ neurogenesis. Our attempts to heterozygously delete *Dgcr8* in ependymal cells failed to reduce the *Dgcr8* transcript levels; therefore, the results of those experiments were inconclusive. We found no noticeable defects in SVZ neurogenesis in adult *Dgcr8*^*+/−*^ mice, which suggested that age-dependent ventricular enlargement in these mice is not caused by structural abnormalities in the SVZ. Together with data on normal ciliary structure in *Dgcr8*^*+/−*^ mice, these data prompted us to think that the slower CBF observed in *Dgcr8*^*+/−*^ mice may arise from functional ciliary abnormalities.

One example of functional defects of motile cilia is primary ciliary dyskinesia (PCD)^91^. PCD is associated with defects in ciliary ultrastructure and/or function of the components of the ciliary axoneme. The clinical features of PCD are present during the neonatal period and characterized by respiratory tract infections, chronic otitis media, situs inversus, male infertility, and in most severe cases, hydrocephalus^91^. Although hydrocephalus is not a consistent feature in PCD, several studies show a link between PCD and hydrocephalus in humans^92–94^ and mouse models^84,85,95^. Despite these numerous studies, it is still not clear whether PCD-related abnormalities in ciliary function cause hydrocephalus. Similarly, we cannot unequivocally claim that slower ciliary beating causes enlarged ventricles in 22q11DS. Nonetheless, our rescue experiments restored the normal levels of both ciliary beating and the ventricular size in 22q11DS mice, and our mimicking experiments resulted in both deceleration of ciliary beating and enlarged LVs in WT mice, suggesting a link between ciliary function and ventricular size. However, the formal causal link between defects in ciliary beating and ventricular enlargement in 22q11DS remains to be established.

Our study reveals a non-neuronal function of Drd1, specifically its role in motile ciliary beating in ependymal cells. The presence of dopamine receptors in ependymal cells and other cells in the subependymal zone and dopaminergic fibers that innervate the ventricular system has been previously reported^67–69^. Dopaminergic signaling in non-neuronal cell populations in the adult subependymal zone may be mediated by paracrine mechanisms^69^. We detected Drd1 expression in the LV wall, including the ependymal cilia. Although we found that both Drd1 and Drd2 are expressed in the LV wall, only Drd1 was detected on the motile cilia. Pharmacologic inhibition of Drd1 accelerates ependymal CBF in mutant mice and brings it to the WT level. This rescue experiment suggests an inverse relation between Drd1 levels in the ependymal cells and their CBF. Consistent with this hypothesis, pharmacological activation of Drd1 reduced ependymal CBF in WT mice. Several studies in different tissues have reported a role of cAMP in mechanisms of ciliary beating with various effects, suggesting that although the structure and function of cilia appears to be conserved across different ciliated tissues, the mechanism regulating this function could be quite diverse^95–97^. Canonical Drd1 signaling activates G_s_ proteins and adenylyl cyclase, which generate cAMP. Consistent with the elevated Drd1 levels, cAMP levels were also higher in the LV wall of *Dgcr8*^*+/−*^ mice. Thus, decreased ependymal CBF caused by *Dgcr8* haploinsufficiency could be explained by elevated Drd1 and the cAMP-dependent pathway.

As part of the Microprocessor complex, Dgcr8 produces miRNA precursors^98,99^. We identified two Drd1-targeting miRNAs that affect ependymal CBF and ventricular volume. *Dgcr8* haploinsufficiency leads to depletion of multiple miRNAs in the LV walls. However, only two miRNAs (miR-382-3p and miR-674-3p) target the *Drd1* 3ʹUTR and regulate its expression. The mimicking and rescuing experiments suggested that the depletion of these two miRNAs is necessary and sufficient to slow ciliary beating and increase ventricular volume in 22q11DS mice. Indeed, depletion of these miRNAs in WT mice increased *Drd1* expression, reduced ependymal CBF, and enlarged the brain ventricles. Conversely, replenishing miR-382-3p or miR-674-3p in ependymal cells eliminated the differences in ependymal cilia beating and ventricular size between WT and 22q11DS mice. These data suggest that miR-382-3p/miR-674-3p directly target *Drd1* to mediate ventricular enlargement in 22q11DS. This hypothesis was confirmed by deleting the unique *Drd1* 13-bp seed site for miR-382-3p/miR-674-3p, which was sufficient to produce ventricular enlargement in LVs. These data strongly suggest that a Dgcr8–miR-382-3p/miR-674-3p–Drd1 mechanism in ependymal cells regulates ciliary beating and the stability of ventricular volumes. Our data show that miR-382-3p and miR-674-3p are highly expressed in the brain, including the LV wall. Yet, it remains to be elucidated how the Dgcr8–miR-382-3p/miR-674-3p–Drd1 pathway affects other brain regions in 22q11DS mice. It is interesting to speculate that these miRNAs might have a differential role in Drd1 signaling in the 22q11DS brain.

The behavioral consequences of ventricular enlargement in 22q11DS or SCZ are unclear. Brain structures adjacent to the ventricles, especially subcortical regions adjacent to the LVs and TV, could be affected by ventricular enlargement. Although ventricular enlargement in SCZ does not correlate with volume diminution in the adjacent brain structures^100^, it could affect neuronal function in those structures. Ventricular enlargement also may affect neuronal functions in subcortical structures (e.g., thalamus, hippocampus, amygdala, and striatum) and potentially explain the diverse symptoms of SCZ (i.e., positive, negative, and cognitive symptoms). Given that ventricular enlargement in 22q11DS mice does not appear until later ages, it could, in theory, contribute to the late onset of at least some symptoms^32,54^. Furthermore, several behavioral abnormalities of 22q11DS mice appear only after 4 months of age, coinciding with the developmental trajectory of ventricular enlargement. For instance, old (>4 months) but not young (<3 months) *Df(16)1/+* mice or similar 22q11DS mouse models perform worse in paired-pulse inhibition, Morris water maze, cued fear conditioning, social memory, and active avoidance tests^48–50,101^. Moreover, the neural circuits associated with these deficits are affected in older but not younger mice^48–50^. For instance, thalamic projections to the auditory cortex or the amygdala become disrupted after 4 months in 22q11DS mice, but 2-month-old mutants have normal synaptic transmission in these circuits^47,48,50^. In this study, we did not investigate the connection between neuroanatomical changes and behavioral deficits in 22q11DS mouse models. Future studies are needed to elucidate those relations.

In summary, our data implicate a Dgcr8–miR-382-3p/miR-674-3p–Drd1 pathogenic mechanism of slower ciliary beating and ventricular enlargement in 22q11DS, a rare disease that substantially increases the risk of several neuropsychiatric disorders including SCZ. Our data indicate that replenishing miR-382-3p or miR-674-3p or inhibiting Drd1 in ependymal cells would be a potential therapeutic avenue for preventing progressive enlargement of brain ventricles, a robust and replicable neuroanatomical feature associated with neuropsychiatric disease.

## Methods

### Animals

Both male and female mice (2–9 months old) were used for all experiments. The generation of *Df(16)1/+*, *Dgcr8*^*+/−*^, *Arl13b*^*eGFP*^, *Foxj1*^*Cre*^, and *Ai14* mouse lines has been reported previously^49,53,58,59,102^. *Df(16)1/+* and *Dgcr8*^*+/−*^ mouse strains were back-crossed onto the C57BL/6J genetic background for at least 10 generations. The care and use of animals were reviewed and approved by the St. Jude Children’s Research Hospital Institutional Animal Care and Use Committee.

### Magnetic resonance imaging

The animal MRI study was performed using a 7T Bruker ClinScan system (Bruker BioSpin MRI GmbH, Germany) equipped with a 12 S gradient coil. A mouse head volume coil (Bruker BioSpin) was used. Animals were anesthetized and maintained with 1.5–2% isoflurane during the experiments. Transverse T2-weighted turbo spin echo images were acquired for volume measurements (Repetition time/Echo time = 3660/50 ms, field of view = 25 × 25 mm, matrix = 320 × 320 pixels, number of averages = 1, thickness = 0.4 mm, scan time = 11.5 min).

### Transmission and scanning electron microscopy

For TEM, 8-month-old WT and *Dgcr8*^*+/−*^ mice were perfused in 4% paraformaldehyde and postfixed in 2.5% glutaraldehyde in 0.1 M sodium cacodylate buffer (Tousimis Research Corp, Rockville, MD or Electron Microscopy Sciences, Hatfield, PA). The samples were postfixed in 2% osmium tetroxide and dehydrated via a graded series of alcohol baths, cleared in propylene oxide, embedded in epon araldite, and polymerized overnight at 70°C. Sections (70-nm thick) were cut on a Leica Ultracut E. The unstained sections were imaged on a JEOL 1200 EX transmission electron microscope with an AMT 2K digital camera.

For scanning electron microscopy, the brains were perfused with super-reagent perfusion wash and super-reagent perfusion fixation (Electron Microscopy Sciences, Hatfield, PA). Freshly collected brains were dissected and fixed with 2.5% glutaraldehyde in 0.1 M sodium cacodylate buffer, pH 7.35 (Tousimis Research Corp, Rockville, MD) and 2% osmium tetroxide, pH 7.35, in 0.1 M sodium cacodylate buffer (Electron Microscopy Sciences) before dehydration in an ethanol series and critically point dried (Tousimis Sandai 790, Tousimis Research Corp). Samples were mounted, coated with gold/palladium, and imaged using a JEOL 7000 field emission gun scanning electron microscope. Cilia length and numbers were measured in a blinded manner (i.e., without prior knowledge of the mouse genotype).

### Immunogold labeling

Mice were perfused with 0.1 M phosphate buffer (pH 7.3) containing 4% paraformaldehyde and 0.1% glutaraldehyde; their brains were then removed and immersed in the same fixative. Brain slices prepared using a vibratome were contrasted with tannic acid and uranyl acetate in 0.1 M sodium acetate, dehydrated in series of solutions with increasing concentrations of ethanol to 100%, infiltrated with resin/ethanol mixtures, and embedded into a modified formulation of Spurr’s resin^103^. The embedded brain slices were sectioned at 80-nm thickness and placed on nickel grids for immunolabeling. The brain sections were sequentially treated with 50 mM glycine in 10 mM PBS (pH 7.4); a blocking solution composed of 5% bovine serum albumin (BSA), 5% normal serum, and 0.1% cold water fish skin gelatin in PBS; and 0.1% BSA-c (Aurion) in PBS. The sections were incubated overnight at 4 °C with anti-DRD1 antibody (Sigma, D2944, 1:10) diluted in 0.1% BSA-c in PBS. After washing with 0.1% BSA-c in PBS, the brain sections were incubated with 12-nm Colloidal Gold AffiniPure donkey anti-rabbit IgG (Jackson Immuno Research, 711-205-152, 1:20) diluted in 0.1% BSA-c in PBS for 2 h at room temperature and washed sequentially with 0.1% BSA-c in PBS, PBS, and water, then allowed to air dry. Samples were examined at 80 kV on an F20 transmission electron microscope (Thermo Fisher Scientific) equipped with an AMT camera system.

### Microbeads-based ependymal flow assay

The LV walls were dissected and pinned in a dish with L-15 Leibovitz media (Thermo Fisher Scientific) at room temperature. Latex beads (1-µm diameter, 5 nL; Sigma-Aldrich, L2778) were released over the dorsal surface of the lateral wall by using a stereotactic injector. Bead movements were recorded using a Rolera-XR high-speed digital camera and an Olympus U-CMAD3 fluorescence microscope using Q-Capture Pro7 imaging software (QImaging, Surrey, BC, Canada), with an acquisition rate of 20 frames/second. Three to five rounds of bead release and imaging were performed for each whole-mount to obtain optimal flow. Negative controls confirmed absent bead flow over the ventricular surface after a 5-min incubation in 70% ethanol.

To trace fluorescent beads in an unbiased manner, we developed a software toolkit in MATLAB (Mathworks, Natwick, MA). In our approach, beads are detected via multiple-scale Laplacian of Gaussian (LoG) filters^104^ as follows:

$$h\left( {i,j} \right) = \frac{{\left( {i^2 + j^2 - 2\sigma ^2} \right)g\left( {i,j} \right)}}{{\sigma ^4{\mathrm{\Sigma }}_m{\mathrm{\Sigma }}_ng\left( {m,n} \right)}},$$

where *g*(*i*,*j*) is a Gaussian (*e*−(*i*^2^+*j*^2^)/2*σ*^2^), *i*,*j* ∈ [−⌈2*σ*⌉, ⌈2*σ*⌉], and *σ* is related to the radius of the bead by *σ* = *r*/√2. In practice, we commonly use filter radii between 3 and 5 pixels. Local optima are extracted from filter-response maps, and a sensitivity threshold is applied to remove weak detections. Next, a nonminima suppression technique discards weak, overlapping detections^105^. Then remaining beads are further pruned in a two-step refinement process: (1) an additional threshold on local maxima from a Gaussian heatmap generated using the spatial location of each detection combines nearby beads into single detections; (2) agglomerative clustering using the complete-linkage criterion over Chebychev distance removes beads that are stationary for too many frames in the image sequence^106^. All thresholds are determined interactively by the user. After detection and refinement, the remaining candidate beads are tracked through time using a Kalman filter with a constant-velocity model^107^. We assume the initial variance of the detections is small (i.e., the LoG filtering/refinement process yields strong detections) and instead increase the motion error of the filter (i.e., the prediction of the Kalman filter is more likely to have an error than is the detection filter due to irregular bead movement and time between image captures). There are additional thresholds on the expected time that a bead is visible in the image (on average); this helps to further prune tracks that are biologically unlikely.

### Ex vivo ciliary beating frequency measurements

For ex vivo measurement of CBF, coronal brain sections (300-µm) were collected in L-15 Leibovitz media (Thermo Fisher Scientific) and placed in 37°C, 95%/5% O_2_/CO_2_ chamber for 15 minutes for incubation. Image sequences of CBF were acquired by linescan analysis using an LD C-Apochromat ×40 objective water-immersion lens (1.1 NA) in LSM780 confocal microscope (Zeiss AxioObserver). Slices were scanned through a line perpendicular to the axis of the cilia in the Xt mode along this line with an acquisition rate of 529 frames/second (512 × 1 frame size, 1.89-ms scan time, 5000 cycles). Imaging was performed within 1 h after the end of the incubation period. Transmitted DIC and fluorescence emitted by GFP or Alexa Fluor 488 were collected with a 488- to 568-nm bandpass filter. Fluorescence excitation and transmitted illumination were provided by a 488-nm argon gas laser. The Drd1 agonist (SKF81297, 10 µM; Tocris, Minneapolis, MN) and antagonist (SCH23390, 10 µM; Tocris) were bath-applied once per slice. Five minutes after the agonist or antagonist was applied, the CBF was measured.

### In vivo ciliary beating frequency measurements

For in vivo measurement of CBF, *Dgcr8*^*+/−*^*;Arl13b*^*eGFP*^ and *Dgcr8*^*+/+*^*;Arl13b*^*eGFP*^ mice of both sexes (aged 6–8 months) were anesthetized with intraperitoneal (IP) injection of ketamine/xylazine (100 mg/kg and 10 mg/kg, respectively) prior to surgery. Toe pinch reflexes were monitored during the procedure, and anesthesia was boosted with IP injection of ketamine (50 mg/kg) when needed. Mice were clamped in position by a headpost; the skin overlaying the left and right frontal and parietal skull bones was removed, and the surface of the skull was cleaned and dried. Two holes (7-mm each) were made in the right frontal skull bone for the placement of stainless steel screws. An aluminum headpost was firmly fastened onto the screws using cyanoacrylate and dental cement. The mouse’s head was positioned at a 45^o^ angle with the left side up. The left temporalis muscle overlying the squamosal bone was then removed and a craniotomy was performed on the left skull using a 0.5-mm drill burr, beginning at the bregma and ~1 mm to the left of the sagittal suture, continuing 5 mm posteriorly down the left parietal bone. The window continued laterally along the left parietal bone and ~2.5 mm down the side of the left squamosal bone. The dura was then removed from the exposed brain surface, and all exposed cortex was aspirated by suction. Blood was constantly removed by aspiration and flushing with saline-15 Leibovitz media until bleeding ceased. With sufficient cortex removed, part of the dorsal and descending hippocampus was exposed, and the LV was visible as an apparent gap between the removed cortex and the hippocampal surface. The mouse was then transferred to the two-photon microscope stage, where it was held firmly via headpost clamp. The skull window was filled with 0.9% saline and positioned under a 25 × water-immersion infrared objective (Olympus, 1.05 NA). GFP^+^ cilia lining the left LV were imaged with 940-nm laser excitation. With a resonant image-scanning resolution of 512 × 512 pixels, regions of interest were chosen to provide a sampling rate of 90–120 Hz. Following the imaging session, mice were immediately euthanized without regaining consciousness. Captured images were stabilized for movement (breathing) artifacts using the Moco ImageJ plugin, and CBF rates were measured manually.

### Quantitative RT-qPCR

Total RNA was isolated from the LV wall (200- to 300-μm-thick slice) using the mirVana microRNA Isolation Kit (Life Technologies, Carlsbad, CA). The iScript kit (Bio-Rad, Hercules, CA) was used to synthesize cDNA from mRNA, and the miRNA First-Strand cDNA Synthesis Kit (Agilent, Santa Clara, CA) was used to synthesize cDNA from mRNA. The qPCR was performed using SYBR Green (Life Technologies). The following forward primers were used for miRNA analysis: mmu-miR-153-5p (5ʹ-TTTGTGACGTTGCAGCT-3ʹ), mmu-miR-382-3p (5ʹ-TCATTCACGGACAACACTTTTT-3ʹ), and mmu-miR-674-3p (5ʹ-CACAGCTCCCATCTCAGAACAA-3ʹ). The universal reverse primer specific to the sequence tag (miRNA First-Strand cDNA Synthesis Kit) was used. The following primers were used for mRNA analysis: *Drd1* forward (5′-ATGGCTCCTAACACTTCTACCA-3′), *Drd1* reverse (5′- GGGTATTCCCTAAGAGAGTGGAC-3′), *Dgcr8* forward (5′-CCACGACCATCCTCAGACATTG-3′), *Dgcr8* reverse (5′-ATGAAAATCTCCCCTCCCCACAGCC-3′). The following loading controls were used: U6 snRNA forward (CGCTTCGGCAGCACATATAC) and U6 snRNA reverse (TTCACGAATTTGCGTGTCAT). Expression levels of *Drd1*, *miR-153-5p*, *miR-382-3p*, and *miR-674-3p* were normalized to the housekeeping gene *U6* for each sample. Samples from each mouse were run in triplicate.

### Generation of miRNA overexpression plasmids

To overexpress the miRNAs of interest, we generated recombinant plasmids by cloning *hsa-miR-30a* chimeric hairpin including sequences of the miRNAs of interest into the 3ʹUTR of *GFP* under the control of the *CAG* promoter by using a previously described strategy^48,108^. The following primers were used: *miR-153-5p-1* (5′-GTACAGCTGTTGACAGTGAGCGACTTTGTGACGTTGCAGCT TGTGAA-3′),

*miR-153-5p-2* (5′-CCATCTGTGGCTTCACAAGCTGCAACGTCACAAAGTCGCTCACTGTCAACAGCT-3′), *miR-153-5p-3* (5′-GCCACAGATGGAGCTGCAACGTCACAAAGCTGCCTACTGCCTCGGAA-3′),

*miR-153-5p-4* (5′-AGCTTTCCGAGGCAGTAGGCAGCTTTGTGACGTTGCAGCT-3′), *miR-382-3p-1* (5′-GTACAGCTGTTGACAGTGAGCGACTCATTCACGGACAACACTTTTT TGTGAA-3′), *miR-382-3p-2* (5′-CCATCTGTGGCTTCACAAAAAAGTGTTGTCCGTGAATGAGTCGCTCACTGTCAACAGCT-3′), *miR-382-3p-3* (5′-GCCACAGATGGAAAAAGTGTTGTCCGTGAATGAGCTGCCTACTGCCTCGGAA-3′), *miR-382-3p-4* (5′-AGCTTTCCGAGGCAGTAGGCAGCTCATTCACGGACAACACTTTTT-3′), *miR-674-3p-1* (5′-GTACAGCTGTTGACAGTGAGCGACCACAGCTCCCATCTCAGAACAATGTGAA-3′), *miR-674-3p-2* (5′-CCATCTGTGGCTTCACATTGTTCTGAGATGGGAGCTGTGGTCGCTCACTGTCAACAGCT-3′),

*miR-674-3p-3* (5′-GCCACAGATGGTTGTTCTGAGATGGGAGCTGTGGCTGCCTACTGCCTCGGAA-3′), and *miR-674-3p-4* (5′-AGCTTTCCGAGGCAGTAGGCAGCCACAGCTCCCATCTCAGAACAA-3′). Recombinant AAVs were generated at the St. Jude Vector Development and Production Core as described previously^48^.

### Generation of miRNA sponge constructs

The *miR-382-3p* and *miR-674-3p* sponges were generated as described previously^48^. Six copies of the following sequences were inserted for the *miR-382-3p* sponge (TTGTTCTGAGATGGGAGCTGTG), the *miR-674-3p* sponge (TCATTCACGGACAACACTTTT), or the scrambled control (GACACTGTGAGCGAAGACATA) into the 3ʹUTR of *GFP* under the control of the *CAG* promoter. Recombinant AAVs were generated at the St. Jude Vector Development and Production Core as described previously^48^.

### Generation of Drd1^Δ7bp^ and Drd1^Δ13bp^ mice

Drd1^Δ7bp^ and Drd1^Δ13bp^ mice were generated using the CRISPR/Cas9 approach. Briefly, C57BL/6 J fertilized zygotes (Jackson Laboratories, Bar Harbor, ME) were cytoplasmically coinjected with 50 ng/µL sgRNA (Synthego), 100 ng/µL SpCas9 in vitro transcribed mRNA (Center for Advanced Genome Engineering at St. Jude), and 50 ng/µL ssODN donor (IDT). Founder mice were genotyped by targeted next-generation sequencing. The following editing construct sequences and relevant primers were used: mDrd1^Δ7bp^.sgRNA (AAUGAGCUGUGCCUCAUCG), mDrd1^Δ7bp^.donor (AGTATCCTCTCTTAAAAAAAAAAAAAAAAGCTCTTTAATGTTAGTGGTAAACTAGCTAATGCCTCATCGTGGAAGTATACACTTCTGTTGTTGGTGGGGGGAATAGAAGAACCCCTTCCC), mDrd1^Δ7bp^.NGS.F including partial Illumina adapters (upper case) (CACTCTTTCCCTACACGACGCTCTTCCGATCTagtcacaggtcacagcagcccctcc), mDrd1^Δ7bp^.NGS.R including partial Illumina adapters (upper case) (GTGACTGGAGTTCAGACGTGTGCTCTTCCGATCTactgttgcaatacccccacccgagg), mDrd1^Δ13bp^.sgRNA (CAGGAUUAAGAUGUGCAUCG), mDrd1^Δ13bp^.donor (TGCTTGAAATGGCTTTCTGAAACAAACAAATGACTGTCCAGGATTAAGATGTGCATCGAGAAAGTCACAGGTCACAGCAGCCCCTCCGATAGTTGGGCTCATCGCTGGTTCTTCATCTGC), mDrd1^Δ13bp^.NGS.F including partial Illumina adapters (upper case) (CACTCTTTCCCTACACGACGCTCTTCCGATCTatggcagaggctttccccgaggcaa), mDrd1^Δ13bp^.NGS.R including partial Illumina adapters (upper case) (GTGACTGGAGTTCAGACGTGTGCTCTTCCGATCTacaaaagtagccccttgagcagccg). Drd1^Δ7bp^ and Drd1^Δ13bp^ lines with corresponding deletions were established. The sequences are as below: for Drd1^Δ7bp^, Drd1 (5ʹ-TGGTAAACTAGCTAATGAGCTGTGCCTCATCGTGGAAGTA-3′) and Drd1^Δ7bp^ (5ʹ-TGGTAAACTAGCTAAT-------GCCTCATCGTGGAAGTA-3′), and for Drd1^Δ13bp^, Drd1 (5ʹ-GATGTGCATCGAGGTGAATGAGCTGTAAAGTCACAGGTCA-3′) and Drd1^Δ13bp^ (5ʹ-GATGTGCATCGAG-------------AAAGTCACAGGTCA-3′).

### Luciferase assay

To test the effect of each miRNA on *Drd1* expression, the 3ʹUTR of the *Drd1* gene was cloned into the 3ʹ end of the *Renilla* luciferase gene contained within the psiCHECK-2 vector (Promega, Madison, WI). The plasmids were transfected into HEK 293 cells (ATCC, CCL-3216) with control *hsa-miR-30a*, *miR-153-5p*, *miR-382-3p*, or the *miR-647-3p OE* plasmid. After 2 days in culture, *Renilla* and Firefly activities were measured using the dual-luciferase reporter assay (Promega) according to the manufacturer’s instructions. The *Renilla* luciferase activity was normalized to Firefly luciferase activity.

### Viral infections and surgery

For in vivo viral injections, young adult mice were anesthetized with isoflurane in pure oxygen (2–3% for induction and 1.0–1.5% for maintenance). Mouse heads were then fixed in a stereotaxic device. Viruses were injected with a metal cannula (33 gauge; Plastics One, Roanoke, VA). An incision was made in the scalp, and a small hole was drilled for the craniotomy. The following stereotaxic coordinates were used for in vivo intracerebroventricle injections: anterior–posterior, –0.22 mm; lateral, +1.0 mm; ventral, –2.0 mm. After injections, incisions were sutured, and mice were allowed to recover before being returned to their holding cages. Experiments were performed ~3 weeks after AAV1 injections.

### Western blotting

Tissue from the LV wall (200- to 300-μm slice) was acutely dissected from the mouse brain and lysed in ice-cold RIPA buffer [50 mM Tris-HCl (pH 7.4), 1% NP-40, 0.25% sodium deoxycholate, 150 mM NaCl, and 1 mM EDTA] that included protease-inhibitor cocktail tablets (Roche, Indianapolis, IN). A total of 25 μg protein was loaded per lane. Sodium dodecyl sulfate/polyacrylamide gel electrophoresis, protein transfer to polyvinylidene difluoride membranes, and Western blotting were performed using standard methods. The following primary antibodies were used: rabbit anti-DRD1 (Abcam, Cambridge, MA; ab40653, 1:500), rabbit anti-DRD2 (Abcam, ab85367, 1:500) and mouse anti–β-actin (Sigma-Aldrich, St. Louis, MO; A5316, 1:10,000). The following secondary antibodies were used: anti-rabbit (LI-COR Biosciences, Lincoln, NE; 926-68021, 1:15,000) and anti-mouse (LI-COR Biosciences, 926-32212, 1:15,000) and antibodies conjugated to IR dye 680 or 800, respectively. Blots were imaged and quantified using the Odyssey CLx infrared imaging system (LI-COR Biosciences).

### Histologic analysis and immunohistochemistry

Mice were deeply anesthetized and intracardially perfused with 4% paraformaldehyde in 0.1 mol/L phosphate buffer (pH 7.4), and brains were fixed overnight. Each brain was sliced (50 µm) coronally with a vibratome (Leica), and the sections including the LV wall were immunolabeled as previously described^109^. The following primary antibodies were used: DRD1 (Sigma-Aldrich, D2944, 1:200), DRD2 (Millipore, Burlington, MA; AB5084p, 1:200), acetylated tubulin (Sigma-Aldrich, T6793, 1:1000), γ-tubulin (Sigma-Aldrich, T5192, 1:1000), β-catenin (BD Transduction Lab, San Jose, CA; 610153, 1:500), GFP (Abcam, ab13970, 1:1000), cleaved caspase-3 (Cell Signaling, Danvers, MA; 9661S, 1:250), Ki67 (Abcam, ab15580, 1:500), BrdU (Abcam, ab6326, 1:200), doublecortin (Abcam, ab18723, 1:1000). Appropriate Alexa dye–conjugated secondary antibodies (Thermo Fisher Scientific, Waltham, MA; 1:1000) were used to detect primary antibody binding. DAPI (Invitrogen, Carlsbad, CA) was used as the nuclear counterstain. For cell proliferation in the SVZ or tracking neuronal migration from the SVZ to the olfactory bulb, BrdU (100 mg/kg) was injected three times at 2-h intervals. Brains were collected either the next day for SVZ analysis or 21 days later for neuronal migration analysis. Measurements of basal body displacement were performed in a blind manner, without prior knowledge of the mouse genotype.

### Super-resolution microscopy

Motile cilia in fixed cortical sections were imaged by structured illumination microscopy (SIM) with a Zeiss ELYRA PS.1 super-resolution microscope (Carl Zeiss MicroImaging, Thornwood, NY) using a ×63 oil objective lens with 1.4 NA at room temperature. Three orientation angles of the excitation grid were acquired for each Z plane, with Z spacing of 110 nm between planes. SIM processing was performed with the SIM analysis module of the Zen 2012 BLACK software (Carl Zeiss MicroImaging) and exported as tiff images.

### Cerebrospinal fluid collection and analysis

For CSF collection, a glass micropipette was inserted into the cisterna magna of anesthetized mice. Collected samples were frozen at −80 °C until analysis. Osmolality was measured by freezing point depression (Advanced Instruments, Norwood, MA) in the Pathology Laboratory at St. Jude.

### miRNA microarray analysis

Total RNA was isolated from the LV wall of 8-month-old WT and *Dgcr8*^*+/−*^ male mice by using the mirVana RNA isolation kit (Life Technologies). Total RNAs (100 ng) were labeled using the miRNA Complete Labeling and Hyb Kit (Agilent), followed by hybridizing to the Mouse miRNA v21 Microarray (Agilent-070155) that contains 4415 unique probes targeting 1,881 mature miRNAs, according to the mouse miRBase version 21.0 (http://www.mirbase.org; June 2014). Microarrays were scanned using an Agilent array scanner (G2565CA) at 3-μm resolution. Microarray data were extracted by Agilent Feature Extraction software (v.10.5.1.1) with the miRNA_107_Sep09 protocol. The data process was performed using Partek software (St. Louis, MO). The miRNA microarray data analysis was performed as described previously^48^. In brief, the signal intensities for each miRNA were summarized after quantile normalization among arrays; the Student’s *t*-test was then performed to determine statistical significance between sets of biological replicates from different experimental groups; and the significant differentially expressed miRNAs were selected through the p-value and fold-change cutoff for the group comparison. The mRNAs targeted by differentially expressed miRNAs were predicted using bioinformatics tools miRWalk and TargetScan. The microarray data have been deposited in the NCBI GEO database under accession number GSE123560.

### Quantification of basal body patch displacement

Quantification of the displacement of the BB patch was performed using previously published methods^60,64^.

### Statistical analyses

All statistics were computed using the Sigma Plot software (Systat Software, Inc., Point Richmond, CA). Differences in mean data were determined by the Student’s *t*-test, Mann-Whitney rank-sum test, Kruskal-Wallis one-way analysis, Watson’s U2 two-sample test, Shapiro-Wilk normality test, or a one-way ANOVA followed by Student–Newman–Keuls post hoc test and were considered significant if the *p*-value of the test result was <0.05.

### Reporting summary

Further information on research design is available in the Nature Research Reporting Summary linked to this article.

## Supplementary information

Supplementary Information

Description of Additional Supplementary Files

Supplementary Movie 1

Supplementary Movie 2

Supplementary Movie 3

Supplementary Movie 4

Supplementary Movie 5

Supplementary Movie 6

Supplementary Movie 7

Reporting Summary

## Data Availability

The microarray data are available in the NCBI GEO here, database under accession number GSE123560. *Drd1*^*Δ7bp+/−*^ and *Drd1*^*Δ13bp+/−*^ mice are available upon request. The source data underlying Figs. 1c, e, 2b, e–h, k, 3m–p, 4d–i, 5b, c, f–i, 6b–d, f–h, 7b, c, e, f, Supplemental Figs. S1a–j, S2b–h, S3b–d, f, S4b–g, i, j, l, S6a, b, S7a, b, S9a–e, g, h, S10a–c, and Supplementary Tables S1–1′, S4–6′ are provided as a Source Data file.

## Source data

Source Data

## Code availability

The software for microbead tracing is available at: https://github.com/dnbornl/beadtracking.
