# Supplementary material for: Schizophrenia-related microdeletion causes defective ciliary motility and brain ventricle enlargement via microRNA-dependent mechanisms in mice
Source: Nat Commun. 2020 Feb 14;11:912. doi: 10.1038/s41467-020-14628-y (PMC7021727; doi:10.1038/s41467-020-14628-y)
Supplement: Supplementary file 1 — Supplementary Information [file 41467_2020_14628_MOESM1_ESM.pdf]

Supplementary Information Titles

Supplementary Item & Number	Title or Caption
Supplementary Figure 1	Neuroanatomical Features in the Brains of *Df(16)1/+* and *Dgcr8*$^{+/–}$ Mice
Supplementary Figure 2	The Effect of Conditional Deletion of *Dgcr8* in Ependymal Cells on Ventricular Enlargement
Supplementary Figure 3	Subventicular Zone Neurogenesis in *Dgcr8*–Conditional Knockout Mice
Supplementary Figure 4	Normal Development of Neural Progenitors in the Subventricular Zone of *Dgcr8*$^{+/–}$ Mice
Supplementary Figure 5	Drd1 Expression in Motile Cilia and the Lack of Drd2 Expression in the LV Wall
Supplementary Figure 6	Conformation of Recombinant miRNA Overexpression and Its Specificity against *Drd1* 3′UTR
Supplementary Figure 7	Validation of the miRNA Sponges
Supplementary Figure 8	In Vivo Expression of AAV1-miRNA-GFP in the Ependymal Cells
Supplementary Figure 9	miRNA Sponges Expressed in the Ependymal Cells Do Not Affect Other Neuroanatomical Features or Planar Polarity
Supplementary Figure 10	Validation of the Overexpression of miRNAs in Vivo
Supplementary Table 1 and 1′	Comparison of ventricular volumes [and ratio of total (ventricles to brain) volumes (VBR)] at two ages in the WT, *Df(16)*1/+, and *Dgcr8*$^{+/–}$ mice
Supplementary Table 2 and 2′	Comparison of ventricular volumes [and ratio of total (ventricles to brain) volumes (VBR)] in 8-month-old *Dgcr8*–conditional knockout mice
Supplementary Table 3	Altered miRNA levels in the lateral ventricle wall of 8-month-old *Dgcr8*$^{+/–}$ mice compared to that of their respective WT littermates
Supplementary Table 4 and 4′	Comparison of ventricular volumes [and ratio of total (ventricles to brain) volumes (VBR)] in 8-month-old WT, *Drd1*$^{\Delta 7bp+/–}$, and *Drd1*$^{\Delta 13bp+/–}$ mice
Supplementary Table 5 and 5′	Comparison of ventricular volumes [and ratio of total (ventricles to brain) volumes (VBR)] in 8-month-old WT mice treated with AAV sponges
Supplementary Table 6 and 6′	Comparison of ventricular volumes [and ratio of total (ventricles to brain) volumes (VBR)] in 8-month-old mice treated with AAV1s that overexpressed microRNAs in ependymal cells
Supplementary Movie 1	Visualization of ependymal flow using microbeads in the LV whole-mounts from 8-month-old WT mice
Supplementary Movie 2	Visualization of ependymal flow using microbeads in the LV whole-mounts from 8-month-old *Dgcr8*$^{+/–}$ mice
Supplementary Movie 3	Visualization of ciliary beating in acute brain slices from 8-month-old WT mice using DIC
Supplementary Movie 4	Visualization of ciliary beating in acute brain slices from 8-month-old *Dgcr8*$^{+/–}$ mice using DIC
Supplementary Movie 5	Fluorescent sequences of cilia over time in the LV whole-mounts from 8-month-old *Dgcr8*$^{+/+}$;*Arl13b*^{eGFP} (WT) mice
Supplementary Movie 6	Fluorescent sequences of cilia over time in the LV whole-mounts from 8-month-old *Dgcr8*$^{+/–}$;*Arl13b*^{eGFP} mice
Supplementary Movie 7	Representative visualization of ciliary beating in vivo in an anesthetized 8-month-old *Dgcr8*$^{+/+}$;*Arl13b*^{eGFP} mouse by using two-photon laser-scanning imaging

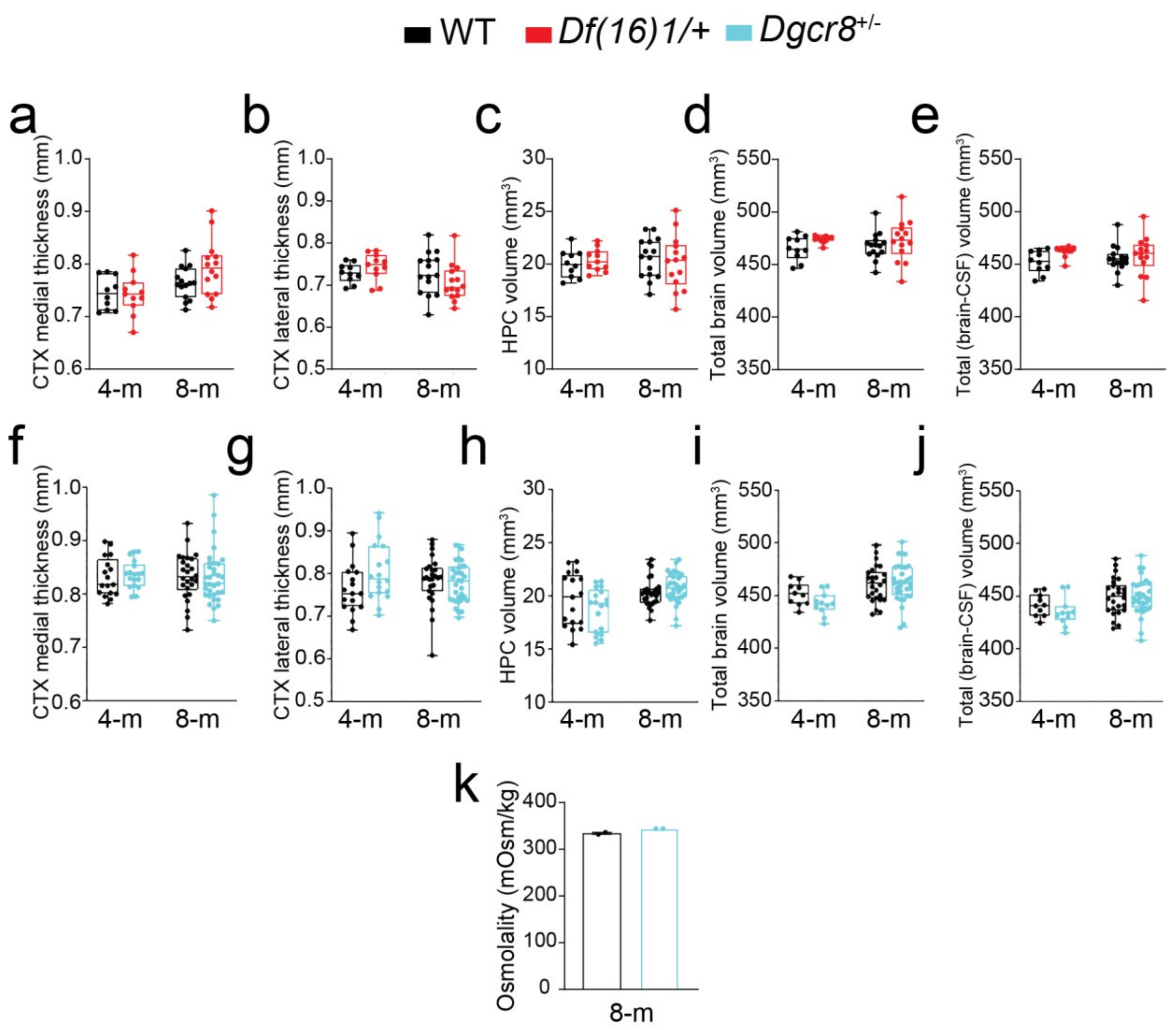

Figure S1. Neuroanatomical Features in the Brains of *Df(16)1/+* and *Dgcr8*$^{+/-}$ Mice. (**a-j**) Cortical (CTX) thickness (**a b**, **f**, **g**), hippocampal (HPC) volume (**c**, **h**), total brain volume with CSF (**d**, **i**) and without CSF (**e**, **j**) in 4- and 8-month-old *Df(16)1/+* (**a-e**) and *Dgcr8*$^{+/-}$ (**f-j**) mice and their respective littermates. (**a-e**) Four-month-old: WT (10 mice) and *Df(16)1/+* (11 mice); two-tailed Student's *t*-test; t_{19}=0.13, *p*=0.89 (**a**); t_{19}=1.25, *p*=0.23 (**b**); t_{19}=0.73, *p*=0.48 (**c**); Mann-Whitney rank-sum test; *U*=30, *p*=0.08 (**d**); *U*=21, *p*=0.02 (**e**); 8-month-old: WT (15 mice) and *Df(16)1/+* (14 mice); t_{27}=1.82, *p*=0.08 (**a**); t_{27}=1.32, *p*=0.20 (**b**); t_{27}=0.51, *p*=0.61 (**c**); t_{27}=0.87, *p*=0.39 (**d**); t_{27}=0.46, *p*=0.65 (**e**). (**f-j**) Four-month-old: WT (10-17 mice) and *Dgcr8*$^{+/-}$ (11-18 mice); t_{33}=0.48, *p*=0.64 (**f**); t_{33}=1.81, *p*=0.08 (**g**); *U*=113, *p*=0.20 (**h**); t_{19}=1.92, *p*=0.07 (**i**); t_{19}=1.22, *p*=0.24 (**j**); 8-month-old: WT (26 mice) and *Dgcr8*$^{+/-}$ (32-33 mice); t_{57}=0.07, *p*=0.95 (**f**); t_{57}=0.43, *p*=0.67 (**g**); t_{57}=1.17, *p*=0.25 (**h**); t_{56}=0.27, *p*=0.79 (**i**); t_{56}=0.07, *p*=0.95 (**j**). (**k**) CSF osmolality in 8-month-old *Dgcr8*$^{+/-}$ (n=14) and WT (n=14) mice. Mann-Whitney rank-sum test; *U*=0, *p*=0.33. Data in this figure and others are shown as either box plots with the median value (**a-j**) or as the mean ± S.E.M (**k**). Source data are provided as a Source Data file.

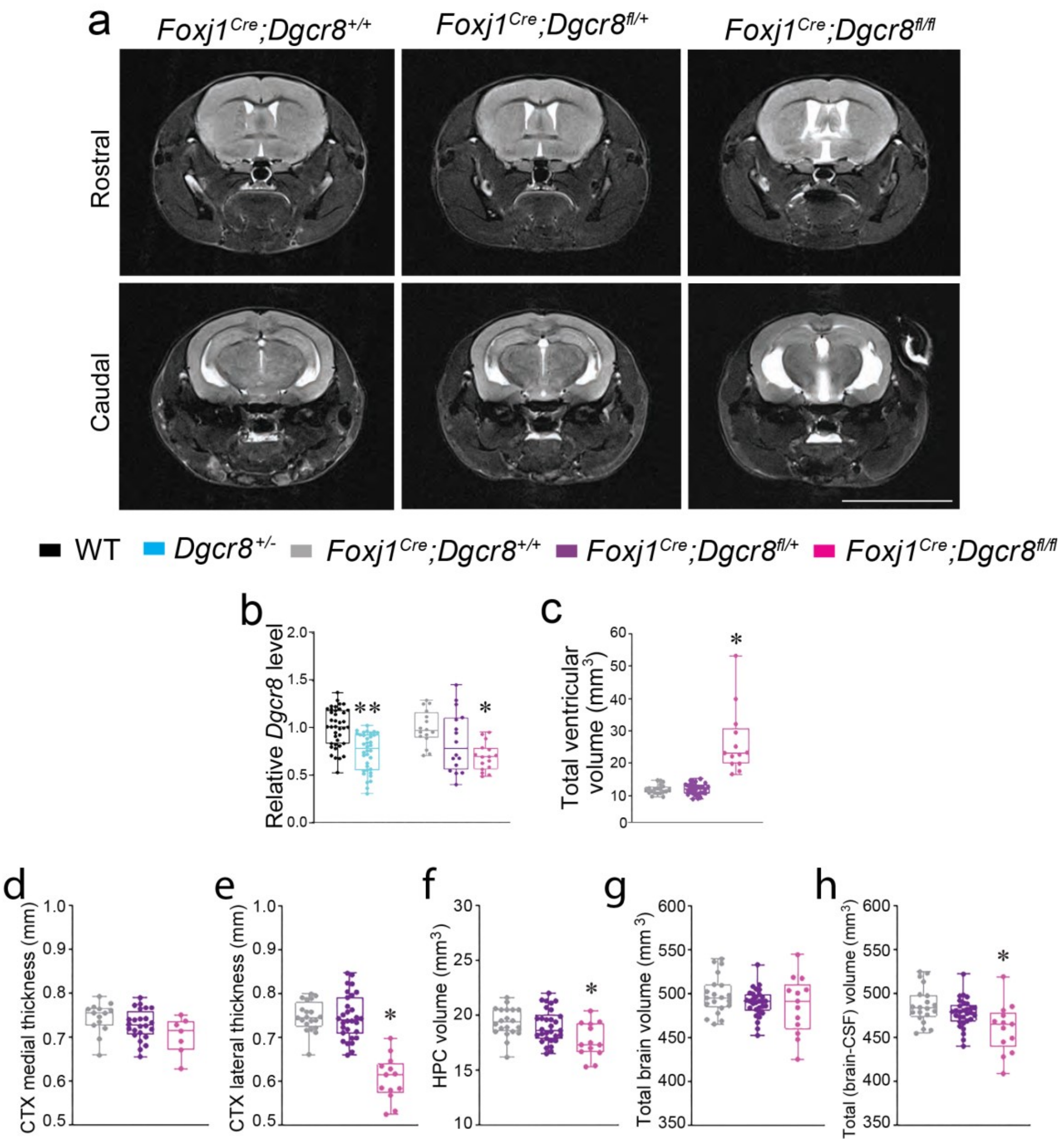

Figure S2. The Effect of Conditional Deletion of *Dgcr8* in Ependymal Cells on Ventricular Enlargement. (**a**) Representative MRIs of the rostral and caudal brains from the *Dgcr8*–conditional knockout mice. (**b**) Normalized *Dgcr8* mRNA levels in the LV wall in the WT (13 mice) and $Dgcr8^{+/-}$ mice (12 mice) (two-tailed Student's *t*-test; t_{73}=5.6, **p <0.001) and in the *Dgcr8*–conditional knockout mice (8 mice per group in each of duplicate experiments; Kruskal-Wallis one-way analysis on ranks: H_2 = 12.99, *p <0.01). (**c**) Total ventricular volume in $Foxj1^{Cre};Dgcr8^{+/+}$(20 mice), $Foxj1^{Cre};Dgcr8^{fl/+}$ (30 mice), and $Foxj1^{Cre};Dgcr8^{fl/fl}$ (13 mice) animals. Kruskal-Wallis one-way analysis on ranks: H_2 = 30.53, *p <0.001). (**d-h**) Cortical (CTX) thickness (Shapiro-Wilk normality test, **d**: One-way ANOVA, F_2=2.8, p=0.07, **e**: F_2=47.47, *p <0.001). (**f**) Hippocampal (HPC) volume (F_2=5.32, *p=0.007). (**g-h**) Total brain volume with CSF (**g**: H_2=1.62, p=0.45) or without CSF (**h**: F_2=6.35, *p=0.003) in *Dgcr8*–conditional knockout mice. All mice were 8 months old. Scale bar, 8 mm. Source data are provided as a Source Data file.

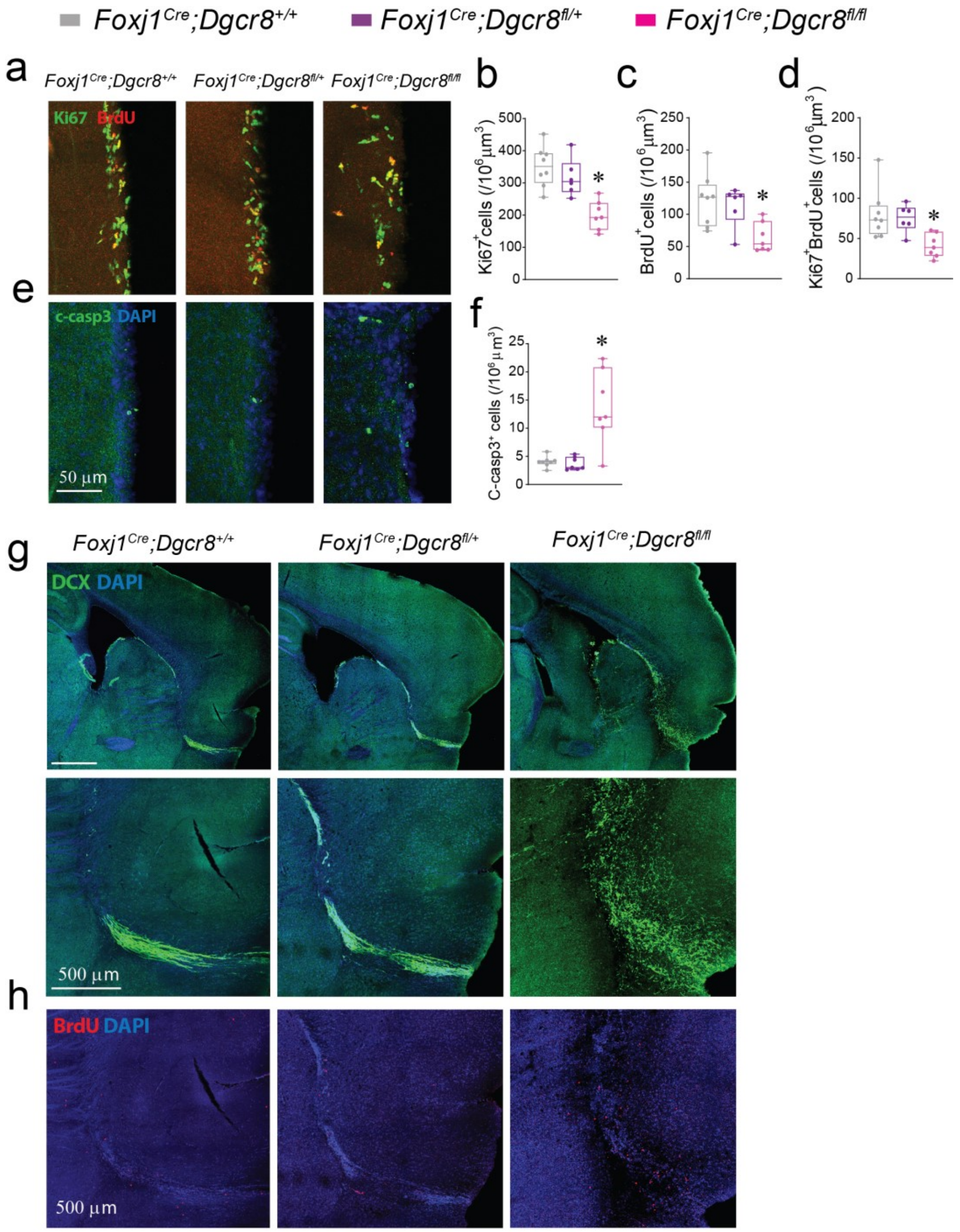

Figure S3. Subventicular Zone Neurogenesis in *Dgcr8*–Conditional Knockout Mice. (**a-f**) SVZ proliferation and apoptosis in *Dgcr8*–conditional knockout mice. (**a**, **e**) Confocal images of BrdU and Ki67 (proliferation markers) and cleaved caspase-3 (an apoptotic marker) staining in the SVZ. (**b-d, f**) Data from *Foxj1*^{Cre}*;Dgcr8*$^{+/+}$ (8 brain sections, 3 mice), *Foxj1*^{Cre}*;Dgcr8*$^{fl/+}$ (6-7 brain sections, 1 mice), and *Foxj1*^{Cre}*;Dgcr8*$^{fl/fl}$ (7 brain sections, 2 mice); Shapiro-Wilk normality test: F_2 = 14.38, *p <0.001 (**b**), F_2 = 6.30, *p=0.008 (**c**); Kruskal-Wallis one-way analysis; H_2=10.31, *p=0.006 (**d**); Kruskal-Wallis one-way analysis; H_2=9.11, *p=0.01 (**f**). (**g**, **h**) Confocal images of doublecortin (DCX) (green) (**g**) and BrdU (red) (**h**) staining, showing SVZ neuronal migratory stream toward the olfactory bulb. Source data are provided as a Source Data file.

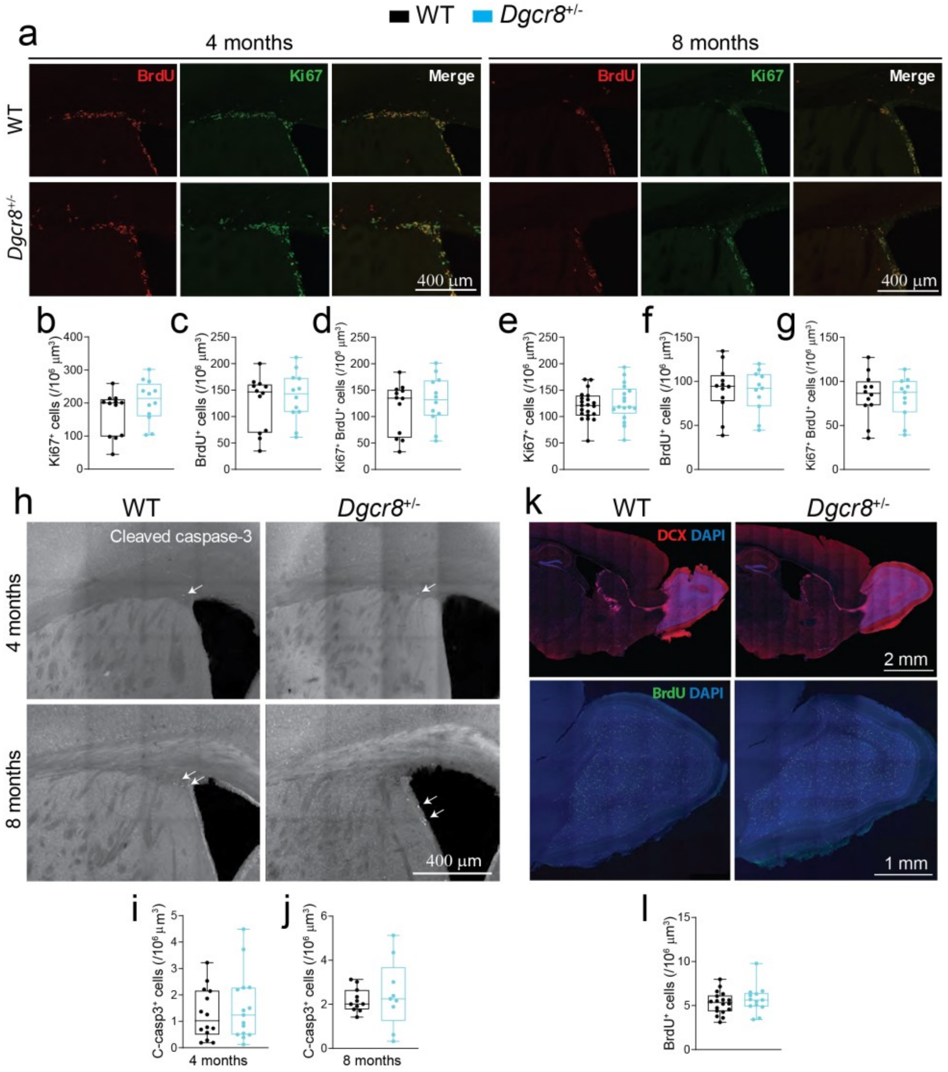

Figure S4. Normal Development of Neural Progenitors in the Subventricular Zone of *Dgcr8*$^{+/-}$ Mice. (**a-g**) Proliferation of neural progenitors in the SVZ. (**a**) Confocal images of BrdU and Ki67 staining in the SVZ of 4- and 8-month-old *Dgcr8*$^{+/-}$ and WT mice. (**b-g**) Average numbers of Ki67^{+} (**b**, **e**), BrdU^{+} (**c**, **f**), and Ki67^{+}/BrdU^{+} (**d**, **g**) cells in the SVZ of 4- and 8-month-old *Dgcr8*$^{+/-}$ and WT mice. (**b-g**) Data from 4-month-old WT (12 brain sections, 4 mice) and *Dgcr8*$^{+/-}$ (12 brain sections, 4 mice); two-tailed Student's *t*-test; t_{22}=1.39, p=0.18 (**b**); t_{22}=0.60, p=0.56 (**c**); t_{22}=0.75, p=0.46 (**d**). Data from 8-month-old WT (12-23 brain sections, 5 mice) and *Dgcr8*$^{+/-}$ (11-17 brain sections, 5 mice) animals; t_{38}=0.69, p=0.50 I; t_{21}=0.23, p=0.82 (**f**); t_{21}=0.26, p=0.80 (**g**). (**h-j**) Apoptotic cell death in the SVZ. Confocal images of cleaved caspase-3 staining (**h**: arrows indicate cleaved caspase-3^{+} cells) and average numbers of caspase-3^{+} cells in the SVZ of 4- and 8-month-old *Dgcr8*$^{+/-}$ and WT mice (**i**, **j**). (**i**) Data from 4-month-old WT (14 brain sections, 4 mice) and *Dgcr8*$^{+/-}$ (15 brain sections, 4 mice) animals; Mann-Whitney rank-sum test; U=96, p=0.71. (**j**) Data from 8-month-old WT (11 brain sections, 3 mice) and *Dgcr8*$^{+/-}$ (9 brain sections, 3 mice) animals; U=43, p=0.65. (**k**) Confocal images of doublecortin (DCX) (red, top) and BrdU (green, bottom) staining. (**l**) Average numbers of BrdU^{+} cells in the olfactory bulb of 8-month-old WT (18 brain sections, 3 mice) and *Dgcr8*$^{+/-}$ (13 brain sections, 3 mice) animals. Two-tailed Student's *t*-test; t_{29}=0.79, p=0.44. Source data are provided as a Source Data file.

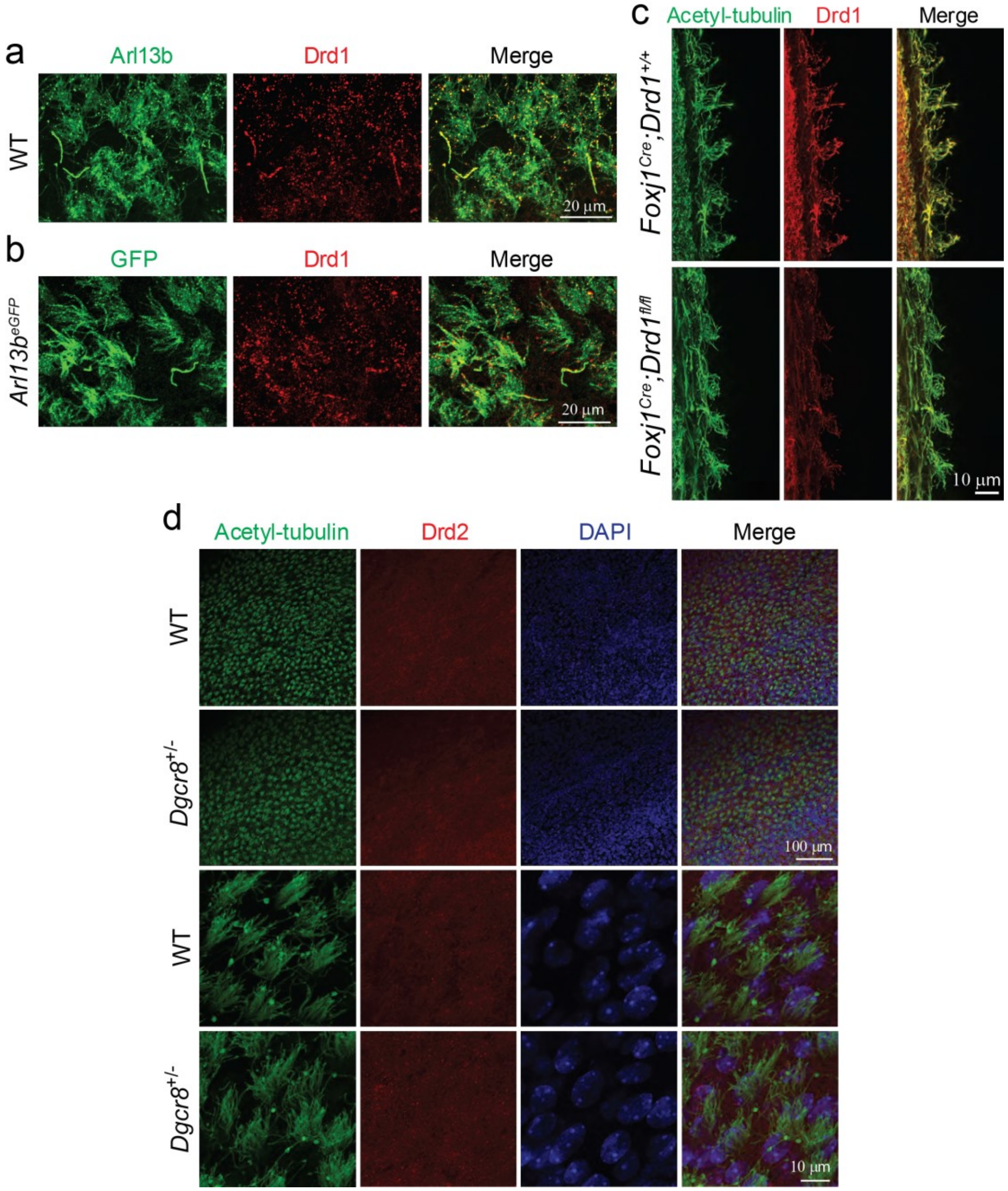

Figure S5. Drd1 Expression in Motile Cilia and the Lack of Drd2 Expression in the LV Wall. (**a, b**) Confocal images show Drd1 expression in the ependymal cilia of whole-mount LV walls from WT (**a**) and *Arl13b*^{eGFP} (**b**) mice. Arl13b (**a**) and GFP (**b**) antibodies are used as markers of motile cilia. (**c**) Super-resolution microscopic images of coronal brain sections from the *Foxj1*^{Cre};*Drd1*$^{+/+}$ (same data as in **Fig. 4b**) and *Foxj1*^{Cre};*Drd1*$^{fl/fl}$ mice show expression of Drd1 in motile cilia. An antibody against acetyl-tubulin is used as a marker of motile cilia. (**d**) Confocal images show a lack of Drd2 expression in the ependymal cells of whole-mount LV walls from WT or *Dgcr8*$^{+/-}$ mice. Acetyl-tubulin antibody is used as a marker of motile cilia, and DAPI stains the nuclei.

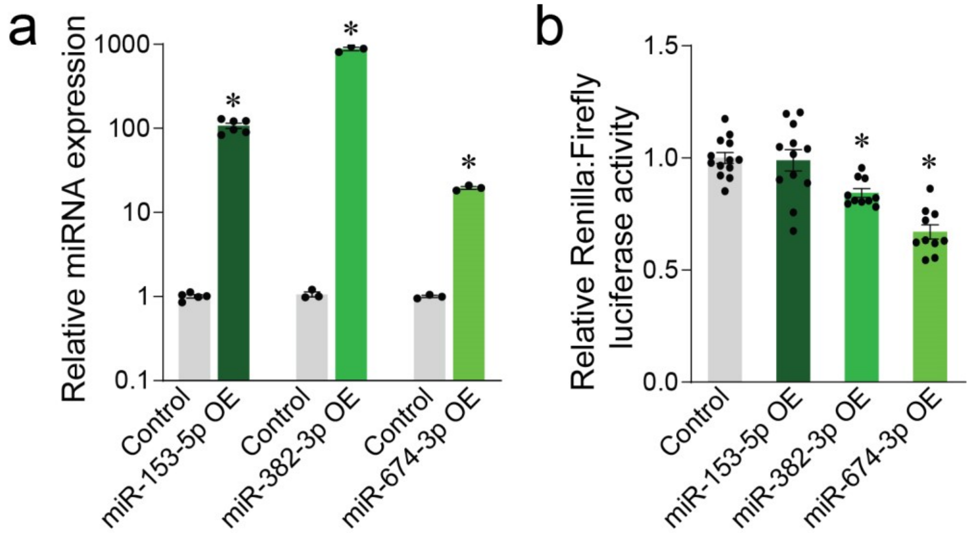

Figure S6. Conformation of Recombinant miRNA Overexpression and Its Specificity against *Drd1* 3′UTR. (**a**) The qRT-PCR analysis of the levels of recombinant miR-153-5p (n=5, control n=6), miR-382-3p (n=3, control n=3), or miR-674-3p (n=3, control n=3) compared to respective controls (Mann-Whitney rank-sum test; miR-153-5p: U=0, *p <0.01, miR-382-3p: two-tailed Student's t-test; t_4=22.58, *p <0.001, miR-674-3p: two-tailed Student's t-test; t_4=24.41, *p <0.001) in the Neuro2A mouse cell line. (**b**) Luciferase assay (one-way ANOVA on Shapiro-Wilk normality test: F_3 = 20.54, *p <0.001) in HEK293 cells co-transfected with *Drd1* 3′UTR–containing luciferase vector and miR-153-5p OE (n=12), miR-382-3p OE (n=10), miR-674-3p OE (n=10), or control vectors (n=13). Source data are provided as a Source Data file.

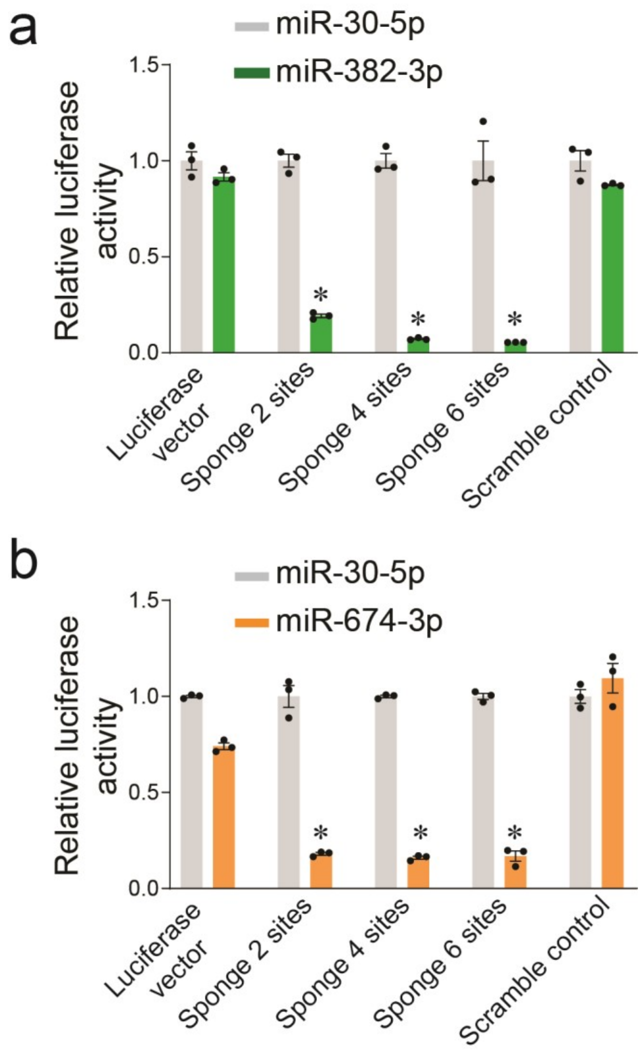

Figure S7. Validation of the miRNA Sponges. (**a**) Luciferase activity measured in Neuro 2a cells transfected with luciferase reporter vector alone, luciferase reporter vector encoding miR-382-3p sponges (two-tailed Student's *t*-test; t_4=1.62, p=0.18) containing two (t_4=23.00, *p <0.001), four (t_4=24.35, *p <0.001), or six (t_4=9.17, *p <0.001) miRNA-binding sites or scrambled sites (t_4=2.36, p=0.08) in the presence of control pGIPZ vector or pGIPZ vector encoding miR-382-3p. Based on these experiments, the miR-382-3p sponge with six seed sites was chosen for in vivo experiments. (**b**) Luciferase activity in Neuro2a cells transfected with luciferase reporter vector alone, luciferase reporter vector encoding miR-674-3p sponges (t_4=14.13, *p <0.001) containing two (t_4=14.25, *p <0.001), four (t_4=80.70, *p <0.001), or six miRNA-binding sites (t_4=26.67, *p <0.001), or scrambled (t_4=1.11, p=0.33) sites in the presence of control pGIPZ vector or pGIPZ vector encoding miR-674-3p. Source data are provided as a Source Data file.

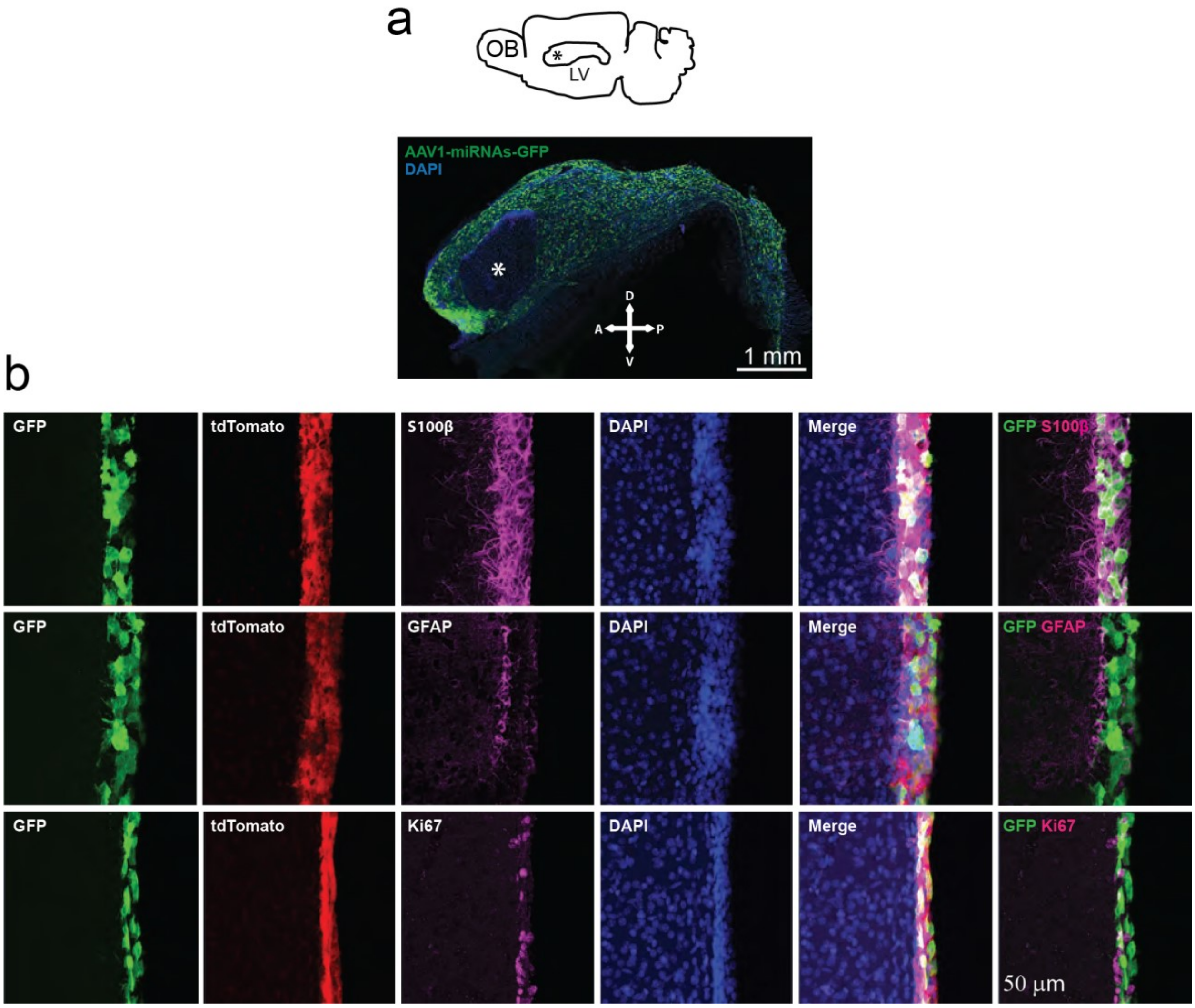

Figure S8. In Vivo Expression of AAV1-miRNA-GFP in the Ependymal Cells. (**a**) Position of the LV wall in the sagittal brain (top) and fluorescent image of GFP in the whole mount excised from the LV wall after injection of AAV1–miRNA-GFP in WT mice (bottom). The adhesion region is denoted by an asterisk. (**b**) Confocal micrographs of AAV1–miRNA-GFP–expressing cell types in the SVZ. GFP-expressing cells that were also tdTomato$^+$ were stained with antibodies against the ependymal cell marker S100β but not with antibodies against the proliferating progenitor markers GFAP or Ki67. Abbreviations: A, anterior; D, dorsal; LV, lateral ventricle; OB, olfactory bulb; P, posterior; V, ventral.

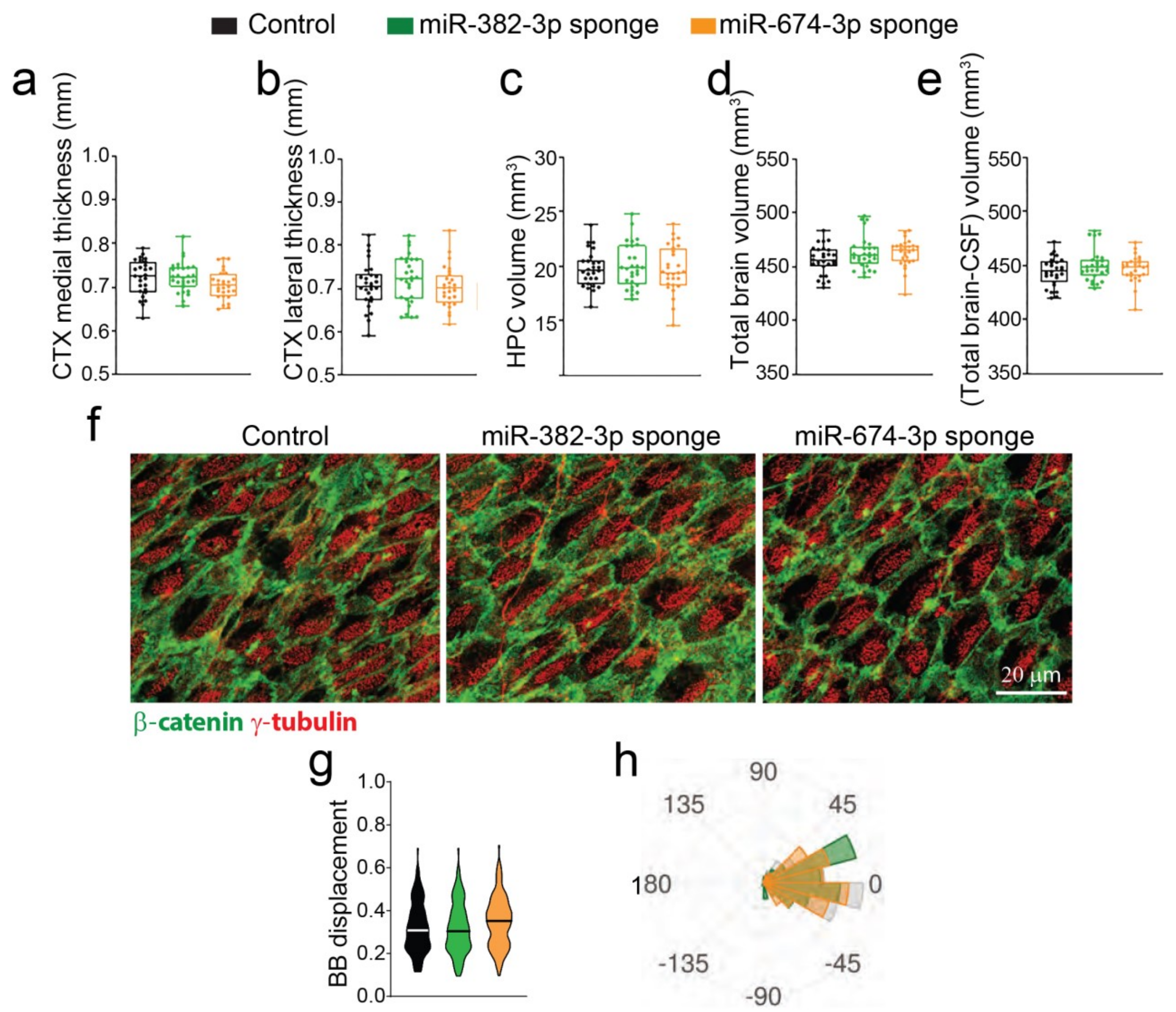

Figure S9. miRNA Sponges Expressed in the Ependymal Cells Do Not Affect Other Neuroanatomical Features or Planar Polarity. (**a-e**) Cortical (CTX) thickness (Shapiro-Wilk normality test, **a**: One-way ANOVA, F_2=2.20, p=0.12, **b**: F_2=0.93, p=0.40), hippocampal (HPC) volume (**c**: F_2=0.24, p=0.79), total brain volume with CSF (**d**: F_2=2.00, p=0.14) or without CSF (**e**: F_2=1.46, p=0.24) in 8-month-old WT mice injected with AAV1–miR-382-3p sponge (28 mice), AAV1–miR-674-3p sponge (28 mice), or AAV1-scramble virus (Control: 27 mice) at 2–3 months of age. (**f**) Confocal images of basal body (BB) patch position (γ-tubulin, red) in ependymal cell junctions (β-catenin, green) in the LV wall of WT mice injected with control AAV1, AAV1–miR-382-3p sponge, or AAV1–miR-674-3p sponge virus. (**g**) Distance of BB displacement from the center of an ependymal cell (**g**: 129 cells from 3 control mice, 131 cells from 3 AAV1–miR-382-3p sponge–infected mice, or 131 cells from 3 AAV1–miR-674-3p sponge–infected mice; Shapiro-Wilk normality test, One-way ANOVA, H_2=4.73, p=0.09). (**h**) Distribution of BB patch plotted on a polar histogram. Average angles of the individual vectors in each imaged section were normalized to 0°, and the distributions of the angles were compared to control (130 cells, 3 mice), AAV1–miR-382-3p sponge– (131 cells, 3 mice), or AAV1–miR-674-3p sponge– (147 cells, 3 mice) infected mice. Watson's U2 two-sample test of homogeneity; t=0.13, p >0.1 for control vs AAV1–miR-382-3p sponge and t=0.05, p >0.1 for control vs AAV1–miR-674-3p sponge. Source data are provided as a Source Data file.

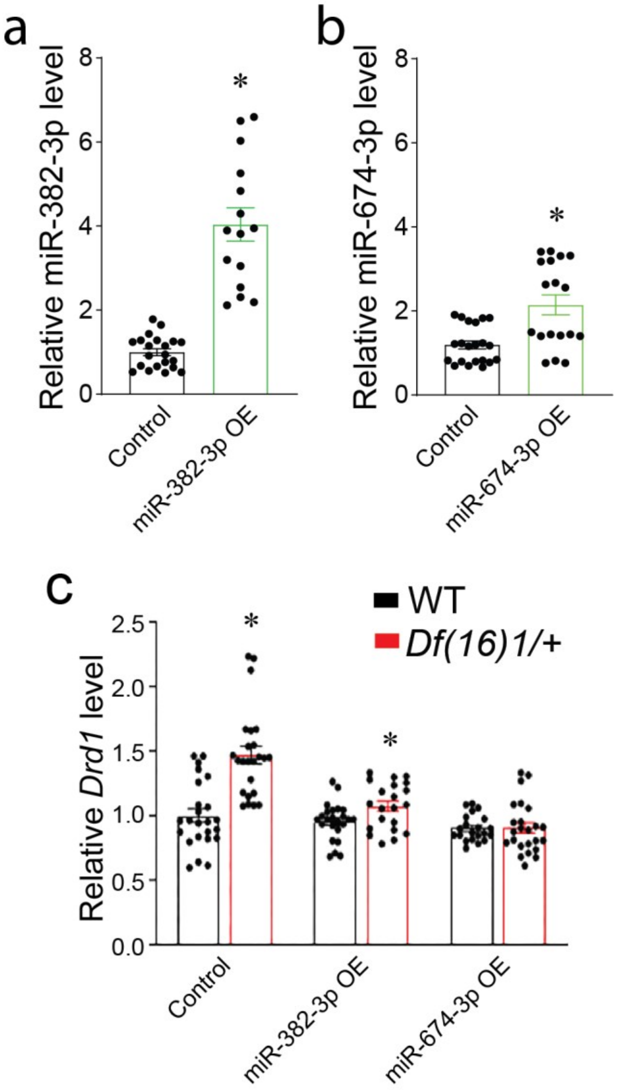

Figure S10. Validation of the Overexpression of miRNAs in Vivo. (**a-b**) The levels of miR-382-3p (**a**) or miR-674-3p (**b**) in the LV wall extracted from WT mice infected with AAV1 expressing scramble-GFP (control), miR-382-3p (miR-382-3p OE), or miR-674-3p (miR-674-3p OE) (Mann-Whitney rank-sum test; miR-382-3p: U=0, *p <0.001, miR-674-3p: U=69, *p <0.001). (**c**) Normalized *Drd1* mRNA in the LV wall extracted from WT or *Df(16)1/+* mice injected with AAV1 expressing control, miR-382-3p OE, or miR-674-3p OE. (Control: 8 mice per group; Mann-Whitney rank-sum test; U=63.5, *p <0.001, miR-382-3p OE: 8 WT mice, 8 *Df(16)/+* mice; Mann-Whitney rank-sum test; U=158, *p <0.05, miR-674-3p OE: 7 WT mice, 8 *Df(16)/+* mice; Mann-Whitney rank-sum test; U=229, p=0.61. Source data are provided as a Source Data file.

Table S1. Comparison of ventricular volumes at two ages in the WT, *Df(16)*1/+, and *Dgcr8*$^{+/-}$ mice

Ventricle Volume[a]	Mean volume ± SD at 4 months				Mean volume ± SD at 8 months			
	**WT (n=10; 2F/8M)**	***Df(16)1/+* (n=11; 2F/9M)**	**% of WT**	***P*-value[b]**	**WT (n=15; 6F/9M)**	***Df(16)1/+* (n=14; 6F/8M)**	**% of WT**	***P*-value[b]**
Fourth	1.11±0.18	0.94±0.12	84±10	**0.01**	0.74±0.26	0.73±0.31	98±42	0.95
Aqueduct	0.53±0.05	0.53±0.09	99±17	0.97	0.55±0.13	0.48±0.12	87±21	0.15
Third	3.34±0.36	3.23±0.65	96±19	1.00	2.65±0.55	3.30±0.72	124±27	**0.007**
Left lateral	3.99±0.81	3.40±0.90	85±22	0.25	3.96±0.78	4.95±1.52	124±38	**0.04**
Right lateral	4.03±0.83	3.52±1.71	87±42	0.40	4.25±0.91	5.24±1.32	123±31	**0.03**
Total	13.27±1.74	11.87±2.52	89±19	0.07	12.36±2.25	14.96±3.07	121±24	**0.01**
	**WT (n=17; 13F/4M)**	***Dgcr8*$^{+/-}$ (n=18; 10M;8F)**	**% of WT**	***P*-value[b]**	**WT (n=26; 6F/20M)**	***Dgcr8*$^{+/-}$ (n=33; 13F/20M)**	**% of WT**	***P*-value[b]**
Fourth	0.88±0.16	0.85±0.14	96±16	0.50	0.84±0.16	0.92±0.15	109±18	**0.05**
Aqueduct	0.51±0.10	0.46±0.12	90±23	0.46	0.52±0.14	0.47±0.13	90±25	0.16
Third	2.38±0.27	2.39±0.41	100±17	0.98	2.43±0.26	2.67±0.31	109±12	**0.002**
Left lateral	3.42±0.66	3.29±1.19	96±34	0.69	3.59±0.78	4.37±1.29	121±35	**0.003**
Right lateral	3.98±1.14	3.59±0.99	90±25	0.30	4.17±0.82	4.62±0.98	110±23	0.06
Total	11.07±1.69	10.15±1.81	91±16	0.13	11.30±1.57	13.04±2.48	115±21	**0.003**
	***Df(16)1/+*[c] (n=11; 2F/9M)**	***Dgcr8*$^{+/-}$[c] (n=18; 10M;8F)**	**% of WT**	***P*-value[b]**	***Df(16)1/+*[c] (n=14; 6F/8M)**	***Dgcr8*$^{+/-}$[c] (n=33; 13F/20M)**	**% of WT**	***P*-value[b]**
Fourth	0.84±0.1	0.96±0.16	114±19	**0.04**	0.99±0.42	1.1±0.18	110±18	0.21
Aqueduct	0.99±0.18	0.91±0.24	91±24	0.31	0.88±0.22	0.9±0.25	102±28	0.77
Third	0.97±0.19	1.0±0.17	103±17	0.59	1.25±0.27	1.1±0.13	87±10	**0.02**
Left lateral	0.85±0.23	0.96±0.35	113±41	0.37	1.25±0.38	1.22±0.36	97±28	0.78
Right lateral	0.87±0.42	0.9±0.25	103±28	0.81	1.23±0.31	1.11±0.24	90±19	0.14
Total	0.89±0.19	0.92±0.16	103±18	0.73	1.21±0.25	1.15±0.22	95±18	0.44

[a] Volumes were measured (in mm^3) using a 7 T Bruker ClinScan system.

[b] *P*-values were calculated by two-tailed Student's *t*-test. Significant p-values are indicated in bold.
[c] Volumes were normalized to the mean volumes of respective WT littermates.
Source data are provided as a Source Data file.

Table S1′. Comparison of the ratio of total (ventricles to brain) volumes (VBR) at two ages in the WT, *Df(16)*1/+, and *Dgcr8*$^{+/-}$ mice

	Mean VBR ± SD at 4 months				Mean VBR ± SD at 8 months			
**VBR**[a]	**WT (n=10; 2F/8M)**	***Df(16)1/+* (n=11; 2F/9M)**	**% of WT**	***P*-value**[b]	**WT (n=15; 6F/9M)**	***Df(16)1/+* (n=14; 6F/8M)**	**% of WT**	***P*-value**[b]
Fourth	0.002±3.8e-4	0.002±2.4e-4	82±9	**0.01**	0.002±5.7e-4	0.002±6.6e-4	97±41	0.88
Aqueduct	0.001±1.1e-4	0.001±2.0e-4	97±17	0.65	0.001±2.7e-4	0.001±2.7e-4	86±22	0.13
Third	0.007±6.5e-4	0.007±1.4e-3	94±19	0.46	0.006±1.1e-3	0.007±1.4e-3	123±25	**0.01**
Left lateral	0.009±1.7e-3	0.007±1.9e-3	83±22	0.10	0.008±1.7e-3	0.01±3.1e-3	123±36	**0.04**
Right lateral	0.009±1.7e-3	0.007±3.7e-3	85±42	0.35	0.009±1.9e-3	0.011±2.8e-3	121±30	**0.03**
Total	0.029±3.5e-3	0.025±5.5e-3	87±19	0.1	0.026±4.7e-3	0.032±6.2e-3	119±23	**0.02**

	**WT (n=10; 8F/2M)**	***Dgcr8*$^{+/-}$ (n=11; 8F/3M)**	**% of WT**	***P*-value**[b]	**WT (n=26; 6F/20M)**	***Dgcr8*$^{+/-}$ (n=33; 13F/20M)**	**% of WT**	***P*-value**[b]
Fourth	0.002±2.8e-4	0.002±3.2e-4	103±17	0.59	0.002±3.6e-4	0.002±3.7e-4	109±19	0.07
Aqueduct	0.001±1.9e-4	0.001±1.8e-4	90±16	0.24	0.001±2.9e-4	0.001±2.9e-4	90±26	0.17
Third	0.005±4.2e-4	0.005±5.8e-4	99±11	0.99	0.005±5.4e-4	0.006±6.5e-4	109±12	**0.002**
Left lateral	0.007±9.6e-4	0.006±1.3e-3	86±19	0.09	0.008±1.8e-3	0.009±2.6e-3	120±34	**0.01**
Right lateral	0.007±1.6e-3	0.007±1.1e-3	94±14	0.49	0.009±1.9e-3	0.01±2.0e-3	110±22	0.08
Total	0.022±3.5e-3	0.021 ±1.8e-3	92±8	0.09	0.025±3.6e-3	0.028±5.1e-3	115±21	**0.003**

	***Df(16)1/+*[c] (n=11; 2F/9M)**	***Dgcr8*$^{+/-}$[c] (n=11; 8F/3M)**	**% of WT**	***P*-value**[b]	***Df(16)1/+*[c] (n=14; 6F, 8M)**	***Dgcr8*$^{+/-}$[c] (n=33; 13F, 20M)**	**% of WT**	***P*-value**[b]
Fourth	0.83±0.1	1.04±0.17	125±20	**0.002**	0.98±0.42	1.1±0.2	112±20	0.19
Aqueduct	0.97±0.18	0.91±0.17	93±17	0.4	0.87±0.23	0.91±0.26	104±30	0.64
Third	0.95±0.2	1±0.12	105±12	0.46	1.23±0.25	1.1±0.12	89±9	**0.02**
Left lateral	0.84±0.22	0.87±0.19	103±23	0.75	1.23±0.37	1.21±0.34	97±27	0.81
Right lateral	0.86±0.42	0.94±0.15	109±17	0.54	1.22±0.31	1.1±0.22	90±18	0.15
Total	0.88±0.19	0.92±0.08	104±9	0.51	1.2±0.23	1.15±0.21	96±17	0.5

[a] Volumes were measured (in mm^3) using a 7 T Bruker ClinScan system.
[b] *P*-values were calculated by two-tailed Student's *t*-test. Significant p-values are indicated in bold.

[c] Volumes were normalized to the mean volumes of respective WT littermates.
Source data are provided as a Source Data file.

Table S2. Comparison of ventricular volumes in 8-month-old *Dgcr8*–conditional knockout mice

Ventricle Volume[a]	Mean volume ± SD						
	***Foxj1**^{Cre}**;Dgcr8**$^{+/+}$* **(n=20; 14F/6M)**	***Foxj1**^{Cre}**;Dgcr8**$^{fl/+}$* **(n=30; 14F/16M)**	**% of WT**	***P*-value[b]**	***Foxj1**^{Cre}**;Dgcr8**$^{fl/fl}$* **(n=13; 3F/10M)**	**% of WT**	***P*-value[b]**
Fourth	0.82±0.17	0.92±0.13	113±15	**0.01**	0.44±0.09	54±10	**<0.001**
Aqueduct	0.64±0.13	0.68±0.16	105±25	0.40	0.53±0.36	82±55	0.22
Third	2.95±0.37	3.01±0.44	102±14	0.58	5.56±1.78	188±60	**<0.001**
Left lateral	3.29±0.79	3.31±0.57	100±17	0.94	NA	NA	**NA**
Right lateral	4.0±0.88	3.87±0.88	96±22	0.60	NA	NA	**NA**
Total	11.92±1.45	12.03±1.68	100±14	0.82	26.56±10.22	222±85	**<0.001**

[a] Volumes were measured (in mm^3) using a 7 T Bruker ClinScan system.
[b] *P*-values were calculated by two-tailed Student's *t*-test. Significant p-values are indicated in bold.
NA, data could not be analyzed due to CSF leakage. Source data are provided as a Source Data file.

Table S2′. Comparison of ventriclar volumes [and ratio of total (ventricles to brain) volumes (VBR)] in 8-month-old *Dgcr8*–conditional knockout mice

Ventricle Volume[a]	Mean volume ± SD						
	***Foxj1**^{Cre}**;Dgcr8**$^{+/+}$* **(n=20; 14F/6M)**	***Foxj1**^{Cre}**;Dgcr8**$^{fl/+}$* **(n=30; 14F/16M)**	**% of WT**	***P*-value[b]**	***Foxj1**^{Cre}**;Dgcr8**$^{fl/fl}$* **(n=13; 3F/10M)**	**% of WT**	***P*-value[b]**
Fourth	0.0024±3.6e-4	0.0019±2.7e-4	114±16	**0.01**	0.001±1.4e-5	55±10	**<0.001**
Aqueduct	0.001±2.7e-4	0.001±3.4e-4	107±26	0.28	0.001±7.7e-4	86±59	0.22
Third	0.006±7.7e-4	0.006±9.4e-4	104±15	0.34	0.011±3.5e-3	192±59	**<0.001**
Left lateral	0.007±1.4e-3	0.007±1.2e-3	102±18	0.62	NA	NA	**NA**
Right lateral	0.008±1.6e-3	0.008±1.9e-3	99±23	0.88	NA	NA	**NA**
Total	0.024±2.3e-3	0.025±3.7e-3	103±15	0.43	0.054±1.9e-2	225±77	**<0.001**

[a] Volumes were measured (in mm^3) using a 7 T Bruker ClinScan system.
[b] *P*-values were calculated by two-tailed Student's *t*-test. Significant p-values are indicated in bold.
NA, data could not be analyzed due to CSF leakage.
Source data are provided as a Source Data file.

Table S3. Altered miRNA levels in the lateral ventricle wall of 8-month-old *Dgcr8*$^{+/-}$ mice compared to that of their respective WT littermates[a]

miRNA ID	*Dgcr8*$^{+/-}$ vs. WT, $\log_2$(FC)	*Dgcr8*$^{+/-}$ vs. WT, *P*-value
miR-374c-5p	-0.7411	3.860E-05
miR-337-5p	-0.6584	3.990E-09
miR-130b-3p	-0.6524	2.970E-05
miR-299b-5p	-0.6510	1.800E-05
miR-337-3p	-0.6427	8.400E-08
miR-329-3p	-0.6317	1.330E-06
miR-672-5p	-0.6314	4.560E-05
miR-409-3p	-0.5983	4.960E-05
miR-674-3p	-0.5980	1.720E-06
miR-379-3p	-0.5889	6.070E-06
miR-873a-5p	-0.5875	2.980E-05
miR-488-3p	-0.5769	2.940E-04
miR-540-5p	-0.5761	2.550E-05
miR-323-3p	-0.5757	2.470E-07
miR-379-5p	-0.5729	5.990E-06
miR-378d	-0.5637	1.080E-06
miR-378b	-0.5582	2.110E-06
miR-382-3p	-0.5511	1.000E-05
miR-544-5p	-0.5479	1.190E-05
miR-382-5p	-0.5265	3.081E-03
miR-411-3p	-0.5244	3.510E-06
miR-582-3p	-0.5198	1.126E-03
miR-467b-5p	-0.5180	1.930E-06
miR-708-3p	-0.5136	6.870E-06
miR-346-5p	-0.5071	3.610E-06
miR-874-3p	-0.4970	1.970E-06
miR-376c-3p	-0.4954	5.040E-05
miR-541-5p	-0.4910	2.840E-05
miR-598-3p	-0.4845	4.623E-03
miR-153-5p	-0.4784	1.650E-07
miR-378a-3p	-0.4762	2.130E-06
miR-192-5p	-0.4760	1.193E-03
miR-409-5p	-0.4759	1.350E-04
miR-592-5p	-0.4725	2.156E-02
miR-340-3p	-0.4680	5.830E-05
miR-378a-5p	-0.4601	9.080E-07
miR-674-5p	-0.4594	1.270E-06
miR-490-3p	-0.4589	4.760E-04

miR-380-3p	-0.4571	3.446E-03
miR-411-5p	-0.4569	6.690E-07
miR-335-3p	-0.4509	1.040E-04
miR-342-5p	-0.4443	3.148E-03
miR-532-5p	-0.4436	7.040E-05
miR-185-5p	-0.4326	1.660E-04
miR-212-5p	-0.4322	9.030E-04
miR-764-3p	-0.4288	3.511E-02
miR-505-5p	-0.4215	5.152E-03
miR-325-3p	-0.4208	9.650E-05
miR-377-3p	-0.4172	1.580E-05
miR-872-5p	-0.4162	1.110E-04
miR-467a-3p	-0.4015	8.680E-05
miR-764-5p	-0.3963	8.540E-04
miR-7118-5p	0.4149	1.866E-02
miR-690	0.4272	6.340E-03
miR-1897-5p	0.4276	1.896E-03
miR-680	0.4285	1.800E-05
miR-6931-5p	0.4313	7.800E-04
miR-7018-5p	0.4319	7.320E-04
miR-1897-3p	0.4362	7.150E-04
miR-3473g	0.4504	1.873E-02
miR-1895	0.4535	2.510E-05
miR-211-3p	0.4605	4.570E-04
miR-5622-3p	0.4787	2.966E-02
miR-7042-5p	0.4934	1.915E-03
miR-7036a-5p	0.5053	3.000E-04
miR-7686-5p	0.5173	3.171E-03
miR-3960	0.5425	6.950E-04
miR-8117	0.5440	9.120E-04
miR-7002-5p	0.5516	7.440E-04
miR-6769b-5p	0.5536	3.223E-03
miR-7047-5p	0.5748	2.489E-03
miR-8110	0.5756	9.110E-04
miR-5126	0.5940	9.900E-04
miR-6997-5p	0.6810	9.220E-03

[a]Only miRNAs with $p < 0.05$ and $\log_2$ fold change (FC) > ± 0.4 are shown. Yellow shading indicates the miRNAs predicted to target the *Drd1* transcript. The microarray data have been deposited in the NCBI GEO database under accession number GSE123560.

Table S4. Comparison of ventricular volumes in 8-month-old WT, *Drd1*$^{\Delta 7bp+/-}$, and *Drd1*$^{\Delta 13bp+/-}$ mice

Ventricle Volume[a]	Mean volume ± SD (mm^3)				Mean volume ± SD (mm^3)			
	**WT (n=11; 6F/5M)**	***Drd1*$^{\Delta 7bp+/-}$ (n=11; 5F/6M)**	**% of WT**	***P*-value[b]**	**WT (n=12; 6F/6M)**	***Drd1*$^{\Delta 13bp+/-}$ (n=11; 1F/10M)**	**% of WT**	***P*-value[b]**
Fourth	0.77±0.13	0.75±0.15	96±19	0.69	0.71±0.11	0.76±0.15	107±20	0.33
Aqueduct	0.64±0.14	0.59±0.17	91±26	0.42	0.50±0.09	0.49±0.13	98±25	0.86
Third	2.81±0.33	2.68±0.31	95±11	0.38	2.65±0.29	2.50±0.27	94±10	0.20
Left lateral	4.56±1.08	4.36±0.78	95±17	0.62	4.10±0.90	4.54±0.97	110±23	0.28
Right lateral	4.64±0.66	4.66±0.98	100±21	0.96	4.03±0.66	4.65±0.51	115±12	**0.02**
LVs (combined)	9.20±1.59	9.02±1.52	98±16	0.79	8.13±0.31	9.19±0.40	112±16	**0.04**
Total	13.67±1.79	13.05±1.84	95±13	0.43 (0.9)[c]	12.23±1.31	13.15±1.47	107±12	0.12 (**0.046**)[c]

[a] Volumes were measured using a 7 T Bruker ClinScan system.
[b] *P*-values were calculated by two-tailed Student's *t*-test. Significant p-values are indicated in bold.
[c] Data in parentheses are *P*-values calculated by two-way ANOVA in 2- 4-, and 8-month-old mice. A significant difference between *Drd1*$^{\Delta 13bp+/-}$ and WT genotypes is indicated in bold.
Abbreviations: F, female; LVs, lateral ventricals; M, male; SD, standard deviation; WT, wild-type
Source data are provided as a Source Data file.

Table S4′. Comparison of ventriclular volumes [and ratio of total (ventricles to brain) volumes (VBR)] in 8-month-old WT, *Drd1*$^{Δ7bp+/–}$, and *Drd1*$^{Δ13bp+/–}$ mice

VBR	Mean VBR ± SD				Mean VBR ± SD			
	**WT (n=11; 6F/5M)**	***Drd1*$^{Δ7bp+/–}$ (n=11; 5F/6M)**	**% of WT**	***P*-value[a]**	**WT (n=12; 6F/6M)**	***Drd1*$^{Δ13bp+/–}$ (n=11; 1F/10M)**	**% of WT**	***P*-value[a]**
Fourth	0.002±2.5e-4	0.002±3.3e-4	97±20	0.72	0.002±2.6e-4	0.002±3.1e-4	109±20	0.27
Aqueduct	0.001±2.8e-4	0.001±3.7e-4	91±27	0.44	0.001±2.1e-4	0.001±2.5e-4	99±22	0.92
Third	0.006±7.0e-4	0.006±5.1e-4	95±8	0.31	0.006±6.2e-4	0.005±4.8e-4	95±8	0.25
Left lateral	0.010±2.4e-3	0.009±1.7e-3	95±18	0.63	0.009±2.1e-3	0.01±2.1e-3	111±24	0.25
Right lateral	0.010±1.6e-3	0.010±1.9e-3	99±18	0.98	0.009±1.4e-3	0.01±1.0e-3	116±11	**0.01**
LVs (combined)	0.019±2.1e-3	0.018±3.0e-3	95±15	0.40	0.018±1.8e-3	0.019±1.3e-3	107±6	**0.049**
Total	0.029±4.2e-3	0.028±3.6e-3	95±12	0.41 (0.677)[b]	0.027±3.0e-3	0.029±3.0e-3	108±11	0.08 (**0.029**)[b]

[a] *P*-values were calculated by two-tailed Student's *t*-test. Significant p-values are indicated in bold.
[b] Data in parentheses are *P*-values calculated by two-way ANOVA in 2- 4-, and 8-month-old mice. A significant difference between *Drd1*$^{Δ13bp+/–}$ and WT genotypes is indicated in bold.
Source data are provided as a Source Data file.

Table S5. Comparison of ventricular volumes in 8-month-old WT mice treated with AAV sponges

**Ventricle Volume**[a]	**Mean volume ± SD after injection of an AAV sponge**						
	**Control (n=27 males)**	***miR-382-3p* (n=28 males)**	**% of WT**	***P*-value**[b]	***miR-674-3p* (n=28 males)**	**% of WT**	***P*-value**[b]
Fourth	0.91±0.21	0.81±0.17	89±18	0.05	0.86±0.20	94±21	0.34
Aqueduct	0.59±0.10	0.54±0.16	92±27	0.12	0.45±0.14	75±23	**<0.001**
Third	2.56±0.40	2.84±0.39	110±15	**0.01**	3.99±1.07	155±41	**<0.001**
Left lateral	3.86±0.80	4.02±0.74	104±19	0.46	4.84±1.17	125±30	**<0.001**
Right lateral	4.11±0.81	4.37±0.61	106±14	0.14	5.14±1.28	125±31	**<0.001**
Total	12.38±1.56	12.85±1.65	103±13	0.28	15.59±2.51	125±20	**<0.001**

[a] Volumes were measured (in mm^3) using a 7 T Bruker ClinScan system.
[b] *P*-values were calculated by two-tailed Student's *t*-test. Significant p-values are indicated in bold.
Source data are provided as a Source Data file.

Table S5′. Comparison of ventricular volumes [and ratio of total (ventricles to brain) volumes (VBR)] in 8-month-old WT mice treated with AAV sponges

**VBR**	**Mean VBR ± SD after injection of an AAV sponge**						
	**Control (n=27 males)**	***miR-382-3p* (n=28 males)**	**% of WT**	***P*-value**[a]	***miR-674-3p* (n=28 males)**	**% of WT**	***P*-value**[a]
Fourth	0.002±4.7e-4	0.0018±3.4e-4	87±17	**0.02**	0.0019±4.3e-4	92±21	0.25
Aqueduct	0.0013±2.4e-4	0.0012±3.3e-4	90±25	0.12	0.001±3.1e-4	74±23	**<0.001**
Third	0.0056±8.1e-4	0.0061±7.5e-4	109±13	**0.02**	0.0086±2.2e-3	153±39	**<0.001**
Left lateral	0.0085±1.7e-3	0.0087±1.5e-3	102±17	0.65	0.0105±2.5e-3	123±29	**0.001**
Right lateral	0.009±1.8e-3	0.0094±1.3e-3	104±13	0.34	0.0111±2.7e-3	123±29	**0.001**
Total	0.027±3.4e-3	0.028±3.1e-3	102±11	0.58	0.034±5.2e-3	124±19	**<0.001**

[a] *P*-values were calculated by two-tailed Student's *t*-test. Significant p-values are indicated in bold.
Source data are provided as a Source Data file.

Table S6. Comparison of ventricular volumes in 8-month-old mice treated with AAV1s that overexpressed microRNAs in ependymal cells

	**Mean volume ± SD after infection with AAV1**										
	***Control* AAV1**			***miR-382-3p OE* AAV1**				***miR-674-3p OE* AAV1**			
**Ventricle Volume[a]**	**WT (n=9; 5F/4M)**	***Df(16)1/+* (n=9; 4F/5M)**	**% of WT and *P*-value[b]**	**WT (n=21; 13F/8M)**	**P-value[b]**	***Df(16)1/+* (n=17; 10F/7M)**	**% of WT and *P*-value[b]**	**WT (n=20; 10F/10M)**	**P-value[b]**	***Df(16)1/+* (n=16; 8F/8M)**	**% of WT and *P*-value[b]**
Fourth	0.94±0.19	0.76±0.18	80±18	0.94±0.18	99±19	0.95±0.22	100±23	0.86±0.27	91±28	0.81±0.19	85±19
			**0.045**		0.97		0.96		0.42		0.09
Aqueduct	0.69±0.18	0.55±0.10	80±14	0.58±0.17	84±25	0.51±0.12	74±17	0.61±0.14	88±20	0.59±0.22	85±32
			0.07		0.14		**0.01**		0.21		0.27
Third	2.81±0.38	3.07±0.52	109±18	2.60±0.34	92±12	2.82±0.59	100±20	2.64±0.46	93±16	2.55±0.49	90±17
			0.25		0.15		0.97		0.33		0.17
Left lateral	3.99±0.63	4.86±1.03	121±25	3.63±0.85	90±21	4.06±1.16	101±29	3.85±0.96	96±22	4.00±0.72	100±17
			**0.046**		0.26		0.88		0.29		0.99
Right lateral	3.98±0.67	4.65±0.56	116±14	3.77±0.66	94±16	4.01±1.29	100±32	4.35±0.92	109±23	3.91±0.93	98±23
			**0.04**		0.43		0.94		0.29		0.84
Total	12.76±1.17	14.14±1.29	107±9	11.79±1.56	92±12	12.58±2.68	98±21	12.56±1.80	98±14	12.18±0.84	95±13
			**0.03**		0.11		0.86		0.77		0.37
	**WT (n=10; 3F/7M)**	***Dgcr8*$^{+/-}$ (n=9; 3F/6M)**	**% of WT and *P*-value[c]**	**WT (n=23; 16F/7M)**	**% change *P*-value[c]**	***Dgcr8*$^{+/-}$ (n=24; 10F/14M)**	**% of WT and *P*-value[c]**	**WT (n=17; 11F/6M)**	**% change *P*-value[c]**	***Dgcr8*$^{+/-}$ (n=17; 13F/4M)**	**% of WT and *P*-value[c]**
Fourth	0.88±0.23	0.99±0.11	112±12	0.87±0.17	97±18	0.84±0.24	94±27	0.83±0.19	94±22	0.84±0.22	94±24
			0.20		0.80		0.59		0.54		0.58
Aqueduct	0.59±0.14	0.54±0.13	90±21	0.56±0.09	94±16	0.55±0.13	92±22	0.61±0.18	102±30	0.61±0.12	102±20
			0.37		0.43		0.44		0.96		0.80
Third	2.96±0.38	3.09±0.44	104±14	2.70±0.60	91±20	2.80±0.61	94±20	2.77±0.39	93±13	2.80±0.46	94±15
			0.50		0.26		0.44		0.24		0.36
Left lateral	3.76±0.39	4.19±0.51	111±13	4.10±1.25	108±33	4.18±1.19	111±31	4.05±0.53	107±14	4.12±0.69	109±18
			0.055		0.74		0.56		0.15		0.15
Right lateral	4.09±0.49	5.10±0.96	124±23	4.30±1.19	105±28	4.52±1.15	110±27	4.38±0.63	107±15	4.33±0.69	105±16
			**0.02**		0.89		0.44		0.22		0.35
Total	12.52±0.94	14.27±1.43	114±11	12.59±3.12	101±23	13.17±2.55	105±20	12.97±1.36	103±10	13.04±1.57	104±12
			**0.005**		0.95		0.72		0.37		0.36

[a] Volumes were measured (in mm^3) using a 7 T Bruker ClinScan system.
[b] *P*-values indicate a comparison of ventricular volume with that measured in WT mice injected with control AAV1, as calculated by two-tailed Student's *t*-test. Significant p-values are indicated in bold.
[c] *P*-values indicate a comparison of ventricular volume with that measured in WT (*Dgcr8*$^{+/+}$) mice injected with control AAV1, as calculated by two-tailed Student's *t*-test. Significant p-values are indicated in bold.
Source data are provided as a Source Data file.

Table S6′. Comparison of ventricular volumes [and ratio of total (ventricles to brain) volumes (VBR)] in 8-month-old mice treated with AAV1s that overexpressed microRNAs in ependymal cells

**VBR**[a]	**Mean VBR ± SD after infection with AAV1**										
	***Control* AAV1**			***miR-382P-3p OE* AAV1**				***miR-674-3p OE* AAV1**			
	**WT (n=9; 5F/4M)**	***Df(16)1/+* (n=9; 4F/5M)**	**% of WT and *P*-value**[b]	**WT (n=21; 13F/8M)**	**% change *P*-value**[b]	***Df(16)1/+* (n=17; 10F/7M)**	**% of WT and *P*-value**[b]	**WT (n=20; 10F/10M)**	**% change *P*-value**[b]	***Df(16)1/+* (n=16; 8F/8M)**	**% of WT and *P*-value**[b]
Fourth	0.002±4.3e-4	0.0016±3.9e-4	79±18	0.002±4.3e-4	98±20	0.002±4.6e-4	98±22	0.002±5.5e-4	89±26	0.002±4.2e-4	84±20
			**0.04**		0.82		0.86		0.29		0.08
Aqueduct	0.002±4.0e-4	0.001±2.3e-4	79±15	0.001±4.0e-4	83±26	0.001±2.7e-4	73±17	0.001±2.9e-4	86±19	0.001±4.9e-4	84±32
			0.06		0.12		**0.01**		0.13		0.24
Third	0.006±8.1e-4	0.007±1.1e-3	108±17	0.006±6.8e-4	90±11	0.006±1.2e-3	98±19	0.006±8.1e-4	91±15	0.005±1.0e-3	89±16
			0.28		0.06		0.80		0.33		0.11
Left lateral	0.009±1.3e-3	0.011±2.0e-3	120±22	0.008±1.3e-3	89±18	0.009±2.4e-3	99±27	0.008±1.8e-3	94±20	0.009±1.6e-3	98±18
			**0.04**		0.13		0.96		0.45		0.86
Right lateral	0.009±1.3e-3	0.010±1.4e-3	116±15	0.008±1.2e-3	92±14	0.009±2.7e-3	98±31	0.009±2.0e-3	107±22	0.008±2.1e-3	96±23
			**0.04**		0.23		0.92		0.41		0.74
Total	0.028±2.2e-3	0.031±2.4e-3	107±8	0.025±2.8e-3	90±10	0.027±5.6e-3	96±20	0.027±3.7e-3	96±13	0.026±3.9e-3	94±14
			**0.02**		**0.02**		0.64		0.44		0.27
	**WT (n=10; 3F/7M)**	***Dgcr8*$^{+/-}$ (n=9; 3F/6M)**	**% of WT and *P*-value**[c]	**WT (n=23; 16F/7M)**	**% change *P*-value**[c]	***Dgcr8*$^{+/-}$ (n=24; 10F/14M)**	**% of WT and *P*-value**[c]	**WT (n=17; 11F/6M)**	**% change *P*-value**[c]	***Dgcr8*$^{+/-}$ (n=17; 13F/4M)**	**% of WT and *P*-value**[c]
Fourth	0.002±4.8e-4	0.002±2.3e-4	112±12	0.002±3.3e-4	99±17	0.002±5.1e-4	95±27	0.002±4.8e-4	91±31	0.002±4.8e-4	95±25
			0.20		0.94		0.64		0.48		0.69
Aqueduct	0.001±2.9e-4	0.001±2.6e-4	90±21	0.001±2.2e-4	95±17	0.001±3.0e-4	93±24	0.001±4.8e-4	100±38	0.001±2.6e-4	103±21
			0.36		0.58		0.46		0.99		0.67
Third	0.006±8.4e-4	0.006±9.2e-4	104±14	0.006±1.2e-3	92±18	0.006±1.2e-3	94±18	0.006±1.6e-3	91±25	0.006±1.1e-3	96±17
			0.53		0.25		0.41		0.31		0.54
Left lateral	0.008±8.4e-4	0.009±1.2e-3	111±14	0.009±2.5e-3	110±31	0.009±2.3e-3	111±28	0.008±2.0e-3	101±25	0.009±1.4e-3	110±18
			0.07		0.33		0.25		0.82		0.10
Right lateral	0.009±9.1e-4	0.011±2.0e-3	124±22	0.009±2.4e-3	106±27	0.010±2.2e-3	110±25	0.009±2.3e-3	101±26	0.009±1.3e-3	106±15
			**0.01**		0.48		0.22		0.84		0.21
Total	0.026±1.8e-3	0.03±3.0e-3	113±11	0.027±5.7e-3	103±21	0.028±4.7e-3	105±17	0.026±6.6e-3	99±24	0.028±3.3e-3	105±12
			**0.005**		0.66		0.36		0.92		0.22

[a] Volumes were measured (in mm^3) using a 7 T Bruker ClinScan system.
[b] *P*-values indicate a comparison of VBR with that measured in WT mice injected with control AAV1, as calculated by two-tailed Student's *t*-test. Significant p-values are indicated in bold.
[c] *P*-values indicate a comparison of VBR with that measured in *Dgcr8*$^{+/+}$ mice injected with control AAV1, as calculated by two-tailed Student's *t*-test. Significant p-values are indicated in bold.
Source data are provided as a Source Data file.

Movies S1-2. Visualization of ependymal flow using microbeads in the LV whole-mounts from 8-month-old WT (**Movie S1**) and *Dgcr8*$^{+/–}$ mice (**Movie S2**). Individual beads are labeled with different colors. Colored traces represent speed and direction of individual bead movements.

Movies S3-4. Visualization of ciliary beating in acute brain slices from 8-month-old WT (**Movie S3**) and *Dgcr8*$^{+/–}$ mice (**Movie S4**) using DIC. More disorganized ciliary beating is observed in *Dgcr8*$^{+/–}$ mice compared to WT littermates.

Movies S5-6. Fluorescent sequences of cilia over time in the LV whole-mounts from 8-month-old *Dgcr8*$^{+/+}$;*Arl13b*^{eGFP} (WT, **Movie S5**) and *Dgcr8*$^{+/–}$;*Arl13b*^{eGFP} (**Movie S6)** mice. More disorganized ciliary beating is observed in *Dgcr8*$^{+/–}$ mice compared to WT littermates.

Movie S7. Representative visualization of ciliary beating in vivo in an anesthetized 8-month-old *Dgcr8*$^{+/+}$;*Arl13b*^{eGFP} mouse by using two-photon laser-scanning imaging.
