# Supplementary material for: Schizophrenia-related microdeletion causes defective ciliary motility and brain ventricle enlargement via microRNA-dependent mechanisms in mice
Source: Nat Commun. 2020 Feb 14;11:912. doi: 10.1038/s41467-020-14628-y (PMC7021727; doi:10.1038/s41467-020-14628-y)
Supplement: Supplementary file 2 — Description of Additional Supplementary Files [file 41467_2020_14628_MOESM2_ESM.docx]

Description of Additional Supplementary Files

**Supplementary Movie 1:** Visualization of ependymal flow using microbeads in the LV whole-mounts from 8-month-old WT mice

**Supplementary Movie 2:**  Visualization of ependymal flow using microbeads in the LV whole-mounts from 8-month-old Dgcr8+/– mice

**Supplementary Movie 3:** Visualization of ciliary beating in acute brain slices from 8-month-old WT mice using DIC

**Supplementary Movie 4:** Visualization of ciliary beating in acute brain slices from 8-month-old Dgcr8+/– mice using DIC

**Supplementary Movie 5:** Fluorescent sequences of cilia over time in the LV whole-mounts from 8- month-old Dgcr8+/+;Arl13beGFP (WT) mice

**Supplementary Movie 6:** Fluorescent sequences of cilia over time in the LV whole-mounts from 8- month-old Dgcr8+/– ;Arl13beGFP mice

**Supplementary Movie 7:** Representative visualization of ciliary beating in vivo in an anesthetized 8- month-old Dgcr8+/+;Arl13beGFP mouse by using two-photon laser-scanning imaging
