# Supplementary material for: Schizophrenia-related microdeletion causes defective ciliary motility and brain ventricle enlargement via microRNA-dependent mechanisms in mice
Source: Nat Commun. 2020 Feb 14;11:912. doi: 10.1038/s41467-020-14628-y (PMC7021727; doi:10.1038/s41467-020-14628-y)
Supplement: Supplementary file 10 — Reporting Summary [file 41467_2020_14628_MOESM10_ESM.pdf]

nature research

Corresponding author(s): Stanislav Zakharenko

Last updated by author(s): 10-10-2019

Reporting Summary

Nature Research wishes to improve the reproducibility of the work that we publish. This form provides structure for consistency and transparency in reporting. For further information on Nature Research policies, see Authors & Referees and the Editorial Policy Checklist.

Statistics

For all statistical analyses, confirm that the following items are present in the figure legend, table legend, main text, or Methods section.

n/a	Confirmed	
☐	☒	The exact sample size (*n*) for each experimental group/condition, given as a discrete number and unit of measurement
☐	☒	A statement on whether measurements were taken from distinct samples or whether the same sample was measured repeatedly
☐	☒	The statistical test(s) used AND whether they are one- or two-sided<br>*Only common tests should be described solely by name; describe more complex techniques in the Methods section.*
☒	☐	A description of all covariates tested
☐	☒	A description of any assumptions or corrections, such as tests of normality and adjustment for multiple comparisons
☐	☒	A full description of the statistical parameters including central tendency (e.g. means) or other basic estimates (e.g. regression coefficient) AND variation (e.g. standard deviation) or associated estimates of uncertainty (e.g. confidence intervals)
☐	☒	For null hypothesis testing, the test statistic (e.g. *F*, *t*, *r*) with confidence intervals, effect sizes, degrees of freedom and *P* value noted<br>*Give P values as exact values whenever suitable.*
☒	☐	For Bayesian analysis, information on the choice of priors and Markov chain Monte Carlo settings
☒	☐	For hierarchical and complex designs, identification of the appropriate level for tests and full reporting of outcomes
☒	☐	Estimates of effect sizes (e.g. Cohen's *d*, Pearson's *r*), indicating how they were calculated

Our web collection on statistics for biologists contains articles on many of the points above.

Software and code

Policy information about availability of computer code

Data collection	Bead tracking software (https://github.com/dnbornl/beadtracking). Laplacian of gaussian filtering (https://doi.org/10.1023/B:VISI.0000029664.99615.94), Kernel-based tracking algorithm (https://doi.org/10.1109/TPAMI.2003.1195991)
Data analysis	SigmaPlot (v12.5), ImageJ, MATLAB R2018a

For manuscripts utilizing custom algorithms or software that are central to the research but not yet described in published literature, software must be made available to editors/reviewers. We strongly encourage code deposition in a community repository (e.g. GitHub). See the Nature Research guidelines for submitting code & software for further information.

Data

Policy information about availability of data

All manuscripts must include a data availability statement. This statement should provide the following information, where applicable:

- Accession codes, unique identifiers, or web links for publicly available datasets
- A list of figures that have associated raw data
- A description of any restrictions on data availability

All data supporting the findings of this study are available upon request.

Field-specific reporting

Please select the one below that is the best fit for your research. If you are not sure, read the appropriate sections before making your selection.

☒ Life sciences ☐ Behavioural & social sciences ☐ Ecological, evolutionary & environmental sciences

Life sciences study design

All studies must disclose on these points even when the disclosure is negative.

Sample size	No sample-size calculation was performed. Sample size was determined based on our experience and the sample size used in similar studies. MRI data for each condition were sampled from 3-33 mice; ependymal flow data from 6 mice; ciliary beating frequency data from 3-6 mice; ciliary length, number, and planar polarity data from 3 mice; RT-qPCR data from 3-8 mice; Western blotting data from 8 mice; cAMP data from 3-4 mice; osmolality data from 14 mice; and SVZ neurogenesis (proliferation, apoptosis, and neuronal migration) data from 3-5 mice.
Data exclusions	Animals in which histological examination showed that viral targeting was in the incorrect location were excluded from analysis.
Replication	All in vitro experiments were repeated at least 2-4 times with similar results. Ex vivo and in vivo experiments were repeated by separate grouping analysis. For example, ciliary beating frequency was measured within each group first and then repeatedly with other groups.
Randomization	Mice in groups were randomly assigned to different experimental conditions.
Blinding	All investigators were blinded to group allocation during data collection and analysis.

Reporting for specific materials, systems and methods

We require information from authors about some types of materials, experimental systems and methods used in many studies. Here, indicate whether each material, system or method listed is relevant to your study. If you are not sure if a list item applies to your research, read the appropriate section before selecting a response.

Materials & experimental systems

n/a	Involved in the study	
☐	☒	Antibodies
☐	☒	Eukaryotic cell lines
☒	☐	Palaeontology
☐	☒	Animals and other organisms
☒	☐	Human research participants
☒	☐	Clinical data

Methods

n/a	Involved in the study	
☒	☐	ChIP-seq
☒	☐	Flow cytometry
☐	☒	MRI-based neuroimaging

Antibodies

Antibodies used

Rabbit polyclonal anti–Dopamine receptor D1, 1:500, Abcam, Cat#ab40653; RRID: AB_732002
Rabbit polyclonal anti–Dopamine receptor D1, 1:5000, Abcam, Cat#ab20066; RRID: AB_445306
Rat monoclonal anti–Dopamine receptor D1, 1:200, Sigma-Aldrich, Cat#D2944; RRID: AB_1840787
Rabbit polyclonal anti–Dopamine D2 receptor, 1:200, Millipore, Cat#ab5084p; RRID: AB_2094980
Rabbit polyclonal anti–Dopamine D2 receptor, 1:500, Abcam, Cat#ab85367; RRID: AB_10674739
Mouse monoclonal anti–Tubulin, Acetylated, 1:1000, Sigma-Aldrich, Cat#T6793; RRID: AB_477585
Rabbit polyclonal anti–γ-Tubulin, 1:1000, Sigma-Aldrich, Cat#T5192; RRID: AB_261690
Mouse monoclonal anti–β-Catenin, 1:500, BD Transduction Lab, Cat#610153; RRID: AB_397554
Chicken polyclonal anti-GFP, 1:1000, Abcam, Cat#ab13970; RRID: AB_300798
Rabbit polyclonal anti–cleaved caspase-3 (Asp175), 1:250, Cell Signaling, Cat#9661; RRID: AB_2341188
Rabbit polyclonal anti-Ki67, 1:500, Abcam, Cat#ab15580; RRID: AB_443209
Rat monoclonal anti-BrdU, 1:200, Abcam, Cat#ab6326; RRID: AB_305426
Rabbit polyclonal anti-Doublecortin, 1:1000, Abcam, Cat#ab18723; RRID: AB_732011
Mouse monoclonal anti–β-Actin, 1:10000, Sigma-Aldrich, Cat#A5316; RRID: AB_476743
Goat-anti–Rabbit IgG (H+L), 1:15000, LI-COR Biosciences Cat#926-68021; RRID: AB_10706309
Donkey-anti–Mouse IgG (H+L), 1:15000, LI-COR Biosciences, Cat#926-32212; RRID: AB_621847
AffiniPure donkey anti-rabbit IgG, 1:20, Jackson Immuno Reserch, Cat#711-205-152; RRID:AB_2340610

Validation

Dopamine receptor D1: Cat#ab40653 was validated in WB, ICC/IF, IHC-P. Reference: PubMed: 29282124. Cat#ab20066 was validated in WB, ELISA, IHC, ICC/IF in mouse, rat, human. Reference PubMed: 27815417. Cat#D2944 was validated in WB, IHC, ICC. Reference: PubMed: 25308843.
Dopamine D2 receptor: Cat#ab5084p was validated in ELISA, IHC, IP, WB in human, mouse, rat, and monkey. Reference: PubMed: 25352792. Cat#ab85367 was validated in WB. Reference: PubMed: 28764937.
Acetylated tubulin, γ-Tubulin, β-Catenin, GFP, cleaved caspase-3, Ki67, Doublecortin, β-Actin, Rabbit IgG (H+L), and Mouse IgG (H+L) were validated previously by multiple studies for use in multiple species (see company website for details).
AffiniPure donkey anti-rabbit IgG was validated in immunogold complexes (see company website for det

Eukaryotic cell lines

Policy information about cell lines

Cell line source(s)	HEK293T
Authentication	Authenticated by sources (ATCC, #CCL-3216)
Mycoplasma contamination	All negative; tested by sources
Commonly misidentified lines (See ICLAC register)	No commonly misidentified cell lines were used

Animals and other organisms

Policy information about studies involving animals; ARRIVE guidelines recommended for reporting animal research

Laboratory animals	Mouse, C57BL/6J background, both male and female were used, and 2-9 months old mice were used for experiments.<br>Mouse: C57BL/6J, the Jackson Laboratory, RRID: IMSR_JAX:000664<br>Mouse: Df(16)1/+, Lindsay et al., 1999, provided by the Illingworth lab<br>Mouse: Dgcr8+/–, Earls et al., 2012<br>Mouse: Dgcr8fl/fl, Yi et al., 2009, provided by the Fuchs lab<br>Mouse: Arl13beGFP, Delling et al., 2013, provided by the Clapham lab<br>Mouse: Foxj1Cre, Zhang et al., 2007, provided by the Holtzman lab<br>Mouse: Ai14, the Jackson Laboratory, RRID: IMSR_JAX:007914<br>Mouse: Drd1(13bp deletion), generated in this paper<br>Mouse: Drd1(7bp deletion), generated in this paper
Wild animals	This study did not involve wild animals.
Field-collected samples	This study did not involve samples collected from the field.
Ethics oversight	The care and use of animals were reviewed and approved by the St. Jude Children's Research Hospital Institutional Animal Care and Use Committee, in accordance with US National Institutes of Health (NIH) guidelines on Care and Use of Laboratory Animals.

Note that full information on the approval of the study protocol must also be provided in the manuscript.

Magnetic resonance imaging

Experimental design

Design type	*Indicate task or resting state; event-related or block design.*
Design specifications	*Specify the number of blocks, trials or experimental units per session and/or subject, and specify the length of each trial or block (if trials are blocked) and interval between trials.*
Behavioral performance measures	*State number and/or type of variables recorded (e.g. correct button press, response time) and what statistics were used to establish that the subjects were performing the task as expected (e.g. mean, range, and/or standard deviation across subjects).*

Acquisition

Imaging type(s)	Structural MRI
Field strength	7T
Sequence & imaging parameters	Turbo spin echo sequence.<br>Transverse: field of view(FOV) 25 x 25 mm, matrix 320 x 320, TR/TE=3800/50 ms, slice thickness 0.4mm<br>Coronal: FOV 25 x 25, matrix 320 x 320, TR/TE = 2200/42 ms, thickness 0.5mm<br>Sagittal: same as Coronal except TR/TE - 3000/39 ms
Area of acquisition	Whole-brain scan was performed, followed by volume analysis of whole brain, cortex, and hippocampus.

Diffusion MRI ☐ Used ☒ Not used

Preprocessing

Preprocessing software	*Provide detail on software version and revision number and on specific parameters (model/functions, brain extraction, segmentation, smoothing kernel size, etc.).*

Normalization	*If data were normalized/standardized, describe the approach(es): specify linear or non-linear and define image types used for transformation OR indicate that data were not normalized and explain rationale for lack of normalization.*
Normalization template	*Describe the template used for normalization/transformation, specifying subject space or group standardized space (e.g. original Talairach, MNI305, ICBM152) OR indicate that the data were not normalized.*
Noise and artifact removal	*Describe your procedure(s) for artifact and structured noise removal, specifying motion parameters, tissue signals and physiological signals (heart rate, respiration).*
Volume censoring	*Define your software and/or method and criteria for volume censoring, and state the extent of such censoring.*

Statistical modeling & inference

Model type and settings	*Specify type (mass univariate, multivariate, RSA, predictive, etc.) and describe essential details of the model at the first and second levels (e.g. fixed, random or mixed effects; drift or auto-correlation).*
Effect(s) tested	*Define precise effect in terms of the task or stimulus conditions instead of psychological concepts and indicate whether ANOVA or factorial designs were used.*

Specify type of analysis: ☐ Whole brain ☐ ROI-based ☐ Both

Statistic type for inference (See Eklund et al. 2016)	*Specify voxel-wise or cluster-wise and report all relevant parameters for cluster-wise methods.*
Correction	*Describe the type of correction and how it is obtained for multiple comparisons (e.g. FWE, FDR, permutation or Monte Carlo).*

Models & analysis

n/a	Involved in the study
☒	☐ Functional and/or effective connectivity
☒	☐ Graph analysis
☒	☐ Multivariate modeling or predictive analysis
